# Supplementary material for: Novel Cecropin-4 Derived Peptides against Methicillin-Resistant Staphylococcus aureus
Source: Antibiotics (Basel). 2021 Jan 1;10(1):36. doi: 10.3390/antibiotics10010036 (PMC7824259; doi:10.3390/antibiotics10010036)
Supplement: Supplementary file 1 [file antibiotics-10-00036-s001.pdf]

Supplementary materials

Novel Cecropin-4 Derived Peptides against Methicillin-Resistant *Staphylococcus aureus*

Jian Peng [1,2#], Biswajit Mishra [1#], Rajamohammed Khader [1], LewisOscar Felix [1] and Eleftherios Mylonakis [1,*]

[1]Infectious Diseases Division, Rhode Island Hospital, Warren Alpert Medical School of Brown University, Providence, RI 02903, USA; jian_peng@brown.edu (J.P.); biswajit_mishra@brown.edu (B.M.); rajamohammed_khader@brown.edu (R.K.); lewis_oscar_felix_raj_lucas@brown.edu (L.F.)

[2]Immune Cells and Antibody Engineering Research Center of Guizhou Province, Key Laboratory of Biology and Medical Engineering, School of Biology and Engineering/School of Basic Medical Sciences, Guizhou Medical University, Guiyang 550025, P.R. China

[#] Both the authors have contributed equally

*Correspondence: emylonakis@lifespan.org

Contents:

Table S1. The physicochemical properties of Cec4 derived peptides

Table S2. The qPCR primers of bacterial virulence genes

Figure S1. The helical wheels of synthetic peptides

Figure S2. The MS and HPLC information of synthetic peptides

Table S1. The physicochemical properties of Cec4 derived peptides

No	Amino acids	Molecular weight (Da)	Theoretical pI	GRAVY	Aliphatic index	Hydrophobicity	Net charge at pH 7.0
Cec4	41	4333.1	10.66	-0.356	95.37	42.2	6
C1	21	2512	11.17	-0.752	92.89	35.47	5
C2	16	1983.44	12.03	-0.9	91.25	31.52	6
C3	16	2068.54	12.03	-0.912	91.25	35.85	6
C4	12	1425.83	11.33	-0.4	121.67	24.37	5
C5	16	2126.58	12.02	-0.725	91.25	43.76	5
C6	16	2184.62	12.02	-0.537	91.25	51.05	4
C7	12	1483.87	11.26	-0.15	121.67	33.41	4
C8	12	1541.91	11.17	0.1	121.67	40.74	3
C9	16	2126.58	12.02	-0.725	91.25	43.29	5
C10	16	2184.62	12.02	-0.537	91.25	51.11	4
C11	12	1483.87	11.26	-0.15	121.67	32.75	4
C12	12	1541.91	11.17	0.1	121.67	40.69	3
C13	16	2255.75	12.02	-0.756	91.25	50.17	5
C14	16	2255.75	12.02	-0.756	91.25	52.97	5
C15	16	2384.91	12.02	-0.787	91.25	60.85	5
C16	16	2326.87	12.03	-0.975	91.25	52.25	6
C17	16	2182.69	12.02	-0.463	115.62	48.46	5
C18	16	2182.69	12.02	-0.463	115.62	51.73	5
C19	16	2238.8	12.02	-0.2	140	57.9	5
C20	16	2180.76	12.03	-0.388	140	49.19	6
C21	16	2442.95	12.02	-0.6	91.25	67.65	4
C22	16	2384.91	12.02	-0.787	91.25	59.08	5
C23	16	2296.84	12.02	-0.013	140	64.91	4
C24	16	2238.8	12.02	-0.2	140	56.25	5

Note: Isoelectric point (pI), Grand average of hydropathicity (GRAVY), amino acid (AA).

Table S2. The qPCR primers of bacterial virulence genes

Gene	Function	Sequence of primer	Size of product (bp)
agrA	Regulator of gene expression	F: 5’ -TGATAATCCTTATGAGGTGCTT-3’<br>R: 5’ -CACTGTGACTCGTAACGAAAA-3’	164
Spa	Surface protein for bacterial aggregation	F: 5’-GCGCAACACGATGAAGCTCAACAA-3’<br>R: 5’-ACGTTAGCACTTTGGCTTGGATCA-3	125
Fnb-A	Surface protein	F: 5’ -ACTTGATTTTGTGTAGCCTTTTT-3’<br>R: 5’ -GAAGAAGCACCAAAAGCAGTA-3’	185
Fnb-B	Surface protein	F: 5’ -CGTTATTTGTAGTTGTTTGTGTT-3’<br>R: 5’ - TGGAATGGGACAAGAAAAAGAA-3’	118
Clf-1	Surface protein	F: 5’ -CGGTTTTGGACTACTCAGCA-3’<br>R: 5’ - GCTACTGCCGATAAACTA-3’	151
srrA	Regulator of gene expression	F: 5’ -AGCATGTGTGGGAGGTATGA-3’<br>R: 5’ -TGCAATCAAATATGATGTGAAGAA-3’	118

Figure S1. The helical wheels of synthetic peptides

C1

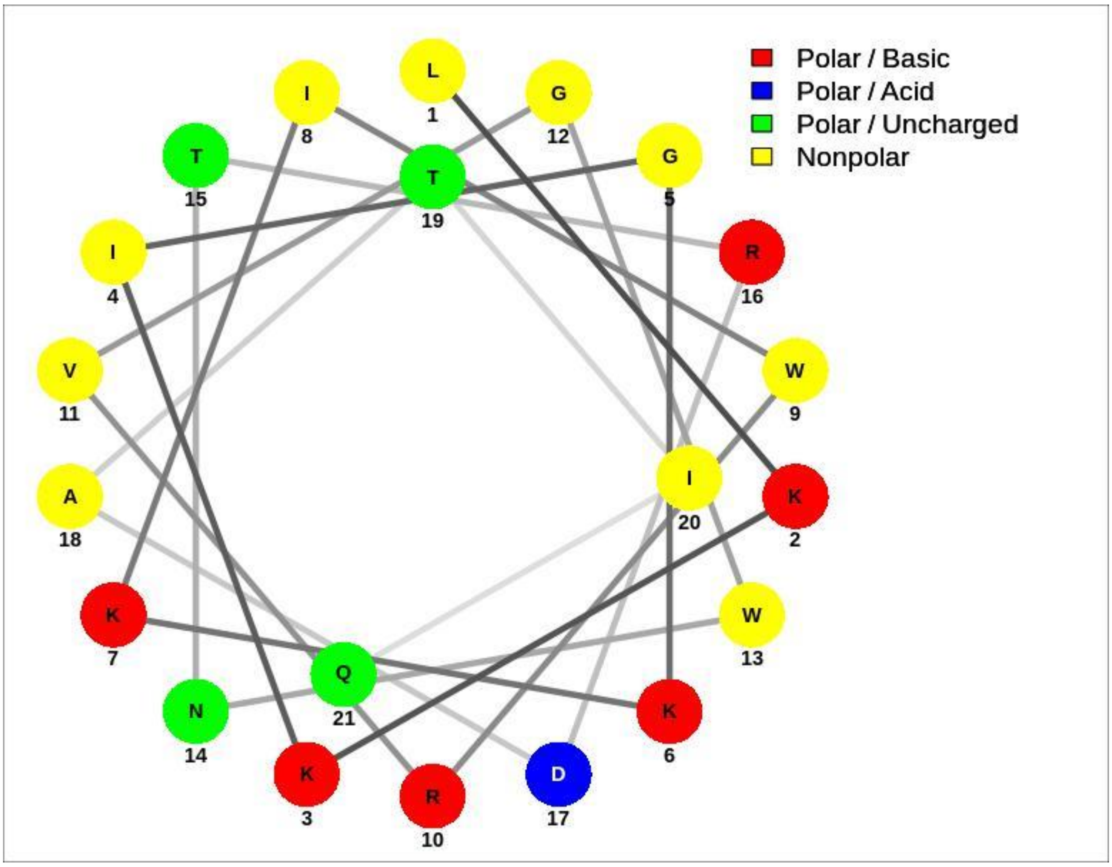

C2

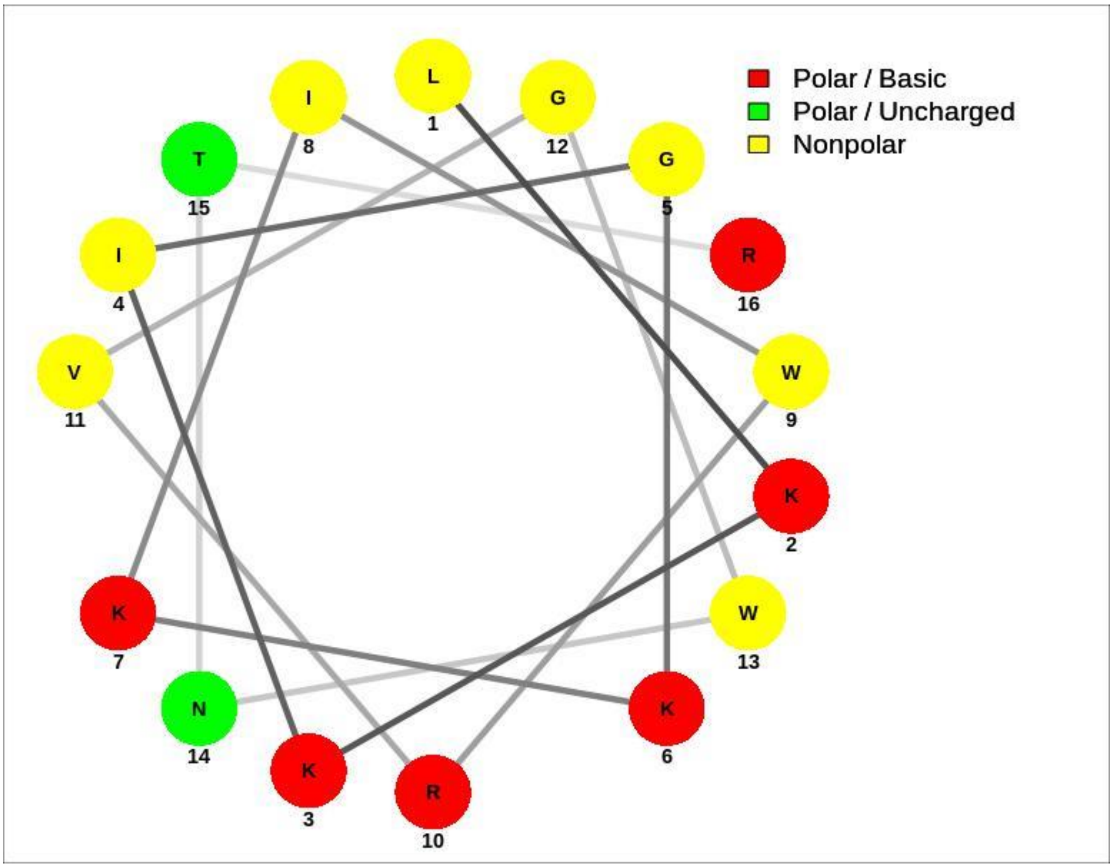

C3

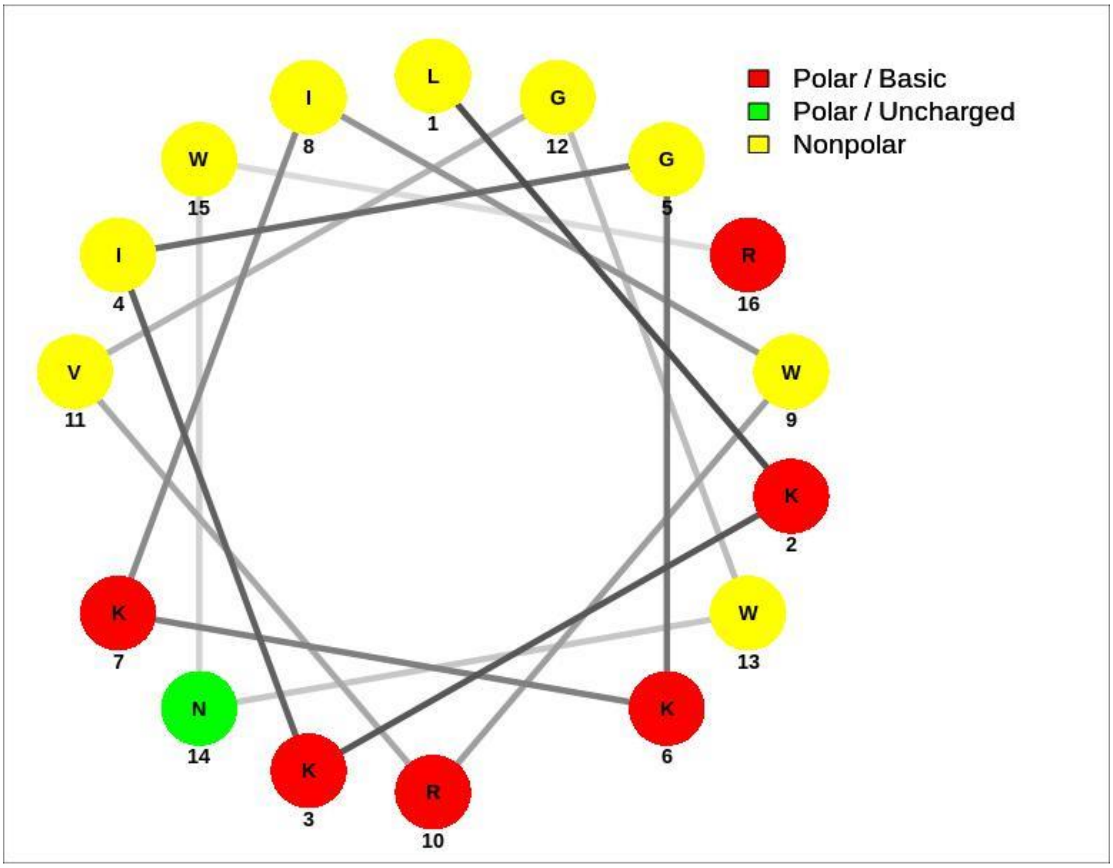
Polar / Basic
Polar / Uncharged
Nonpolar
L 1
K 2
K 3
I 4
G 5
K 6
K 7
I 8
W 9
R 10
V 11
G 12
W 13
N 14
W 15
R 16

C4

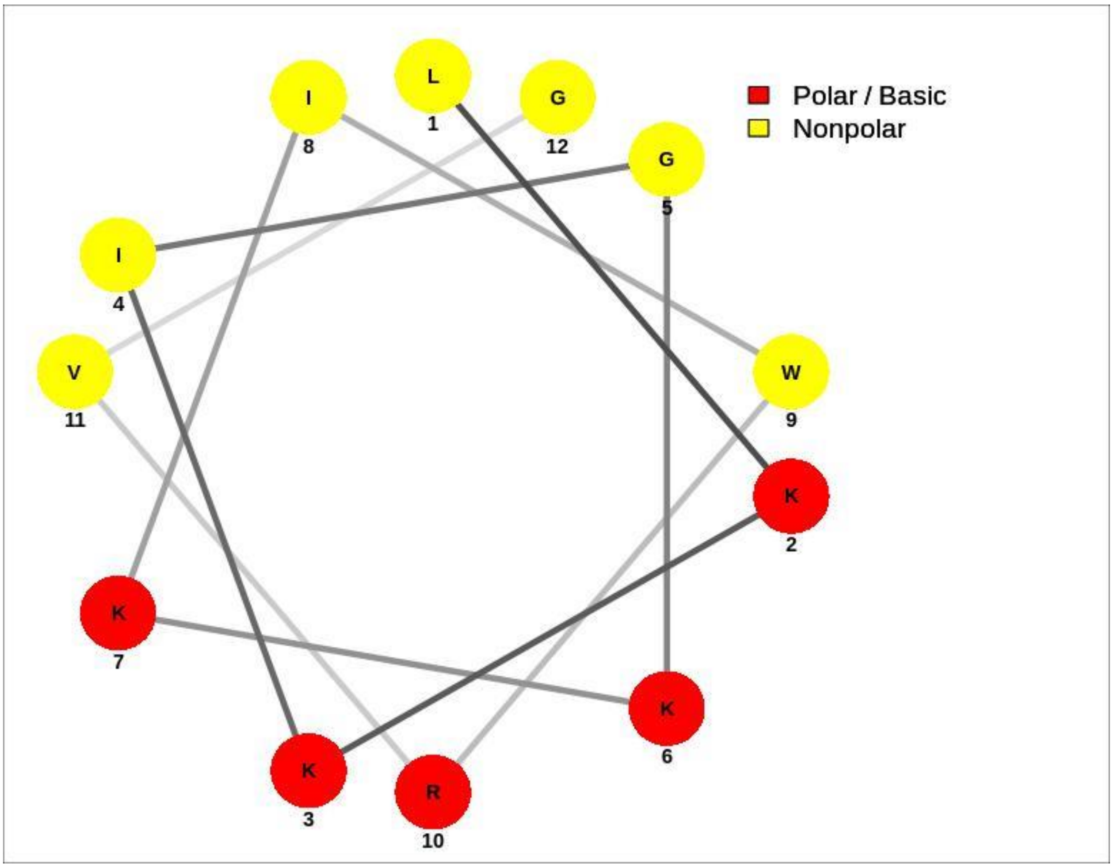
Polar / Basic
Nonpolar
L 1
K 2
K 3
I 4
G 5
K 6
K 7
I 8
W 9
R 10
V 11
G 12

C5

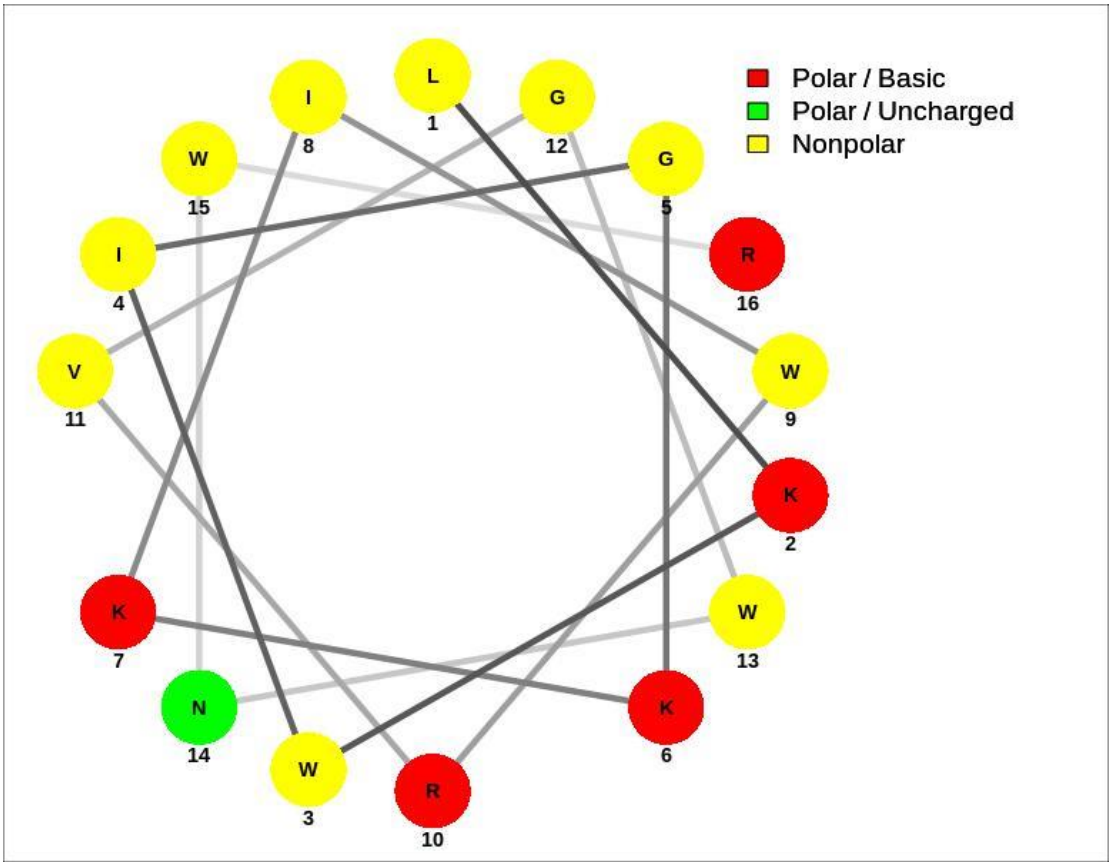
L 1
G 12
G 5
R 16
W 9
K 2
W 13
K 6
R 10
W 3
N 14
K 7
V 11
I 4
W 15
I 8
Polar / Basic
Polar / Uncharged
Nonpolar

C6

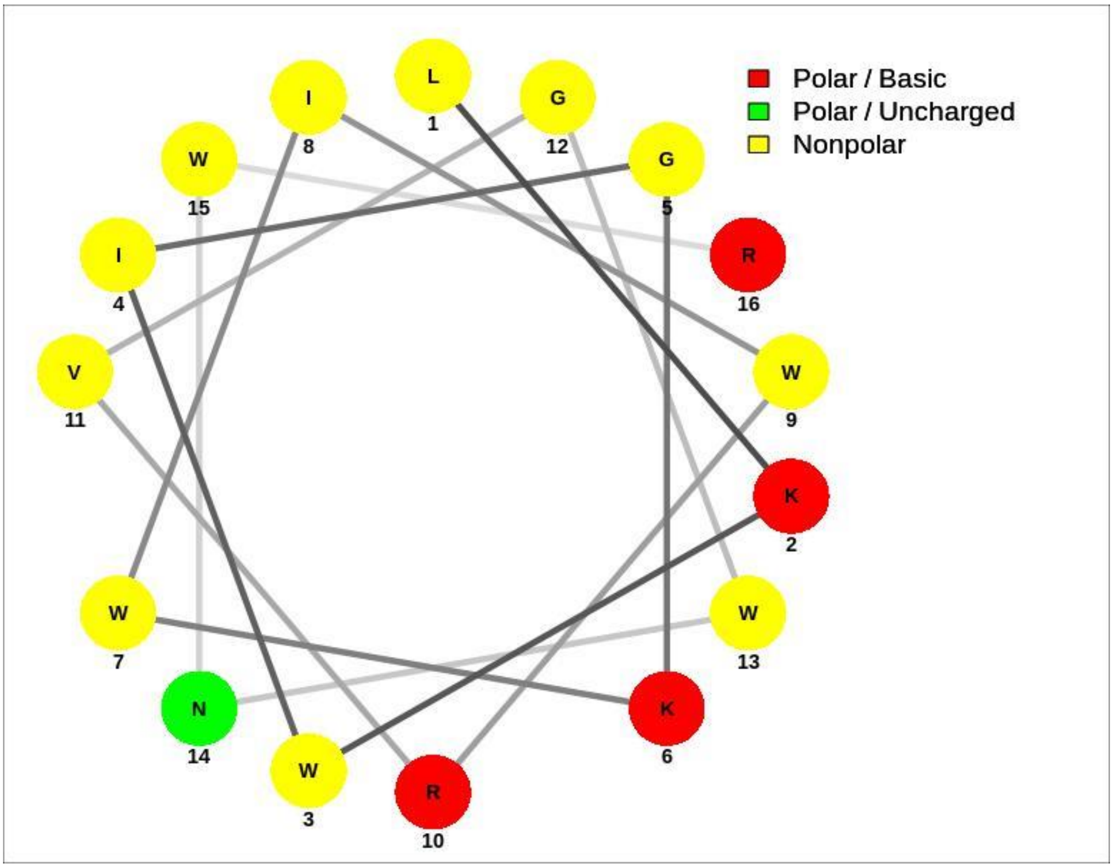
L 1
G 12
G 5
R 16
W 9
K 2
W 13
K 6
R 10
W 3
N 14
W 7
V 11
I 4
W 15
I 8
Polar / Basic
Polar / Uncharged
Nonpolar

C7

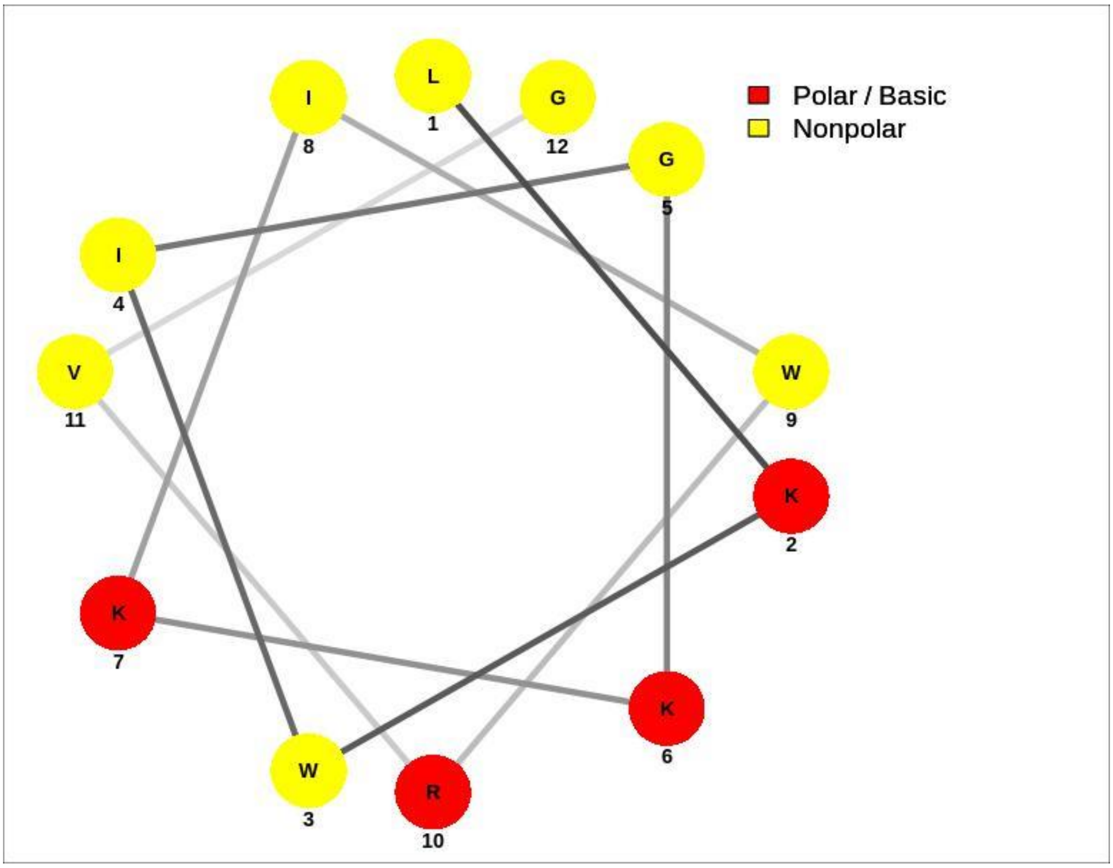
L
1
G
12
G
5
W
9
K
2
K
6
R
10
W
3
K
7
V
11
I
4
I
8
Polar / Basic
Nonpolar

C8

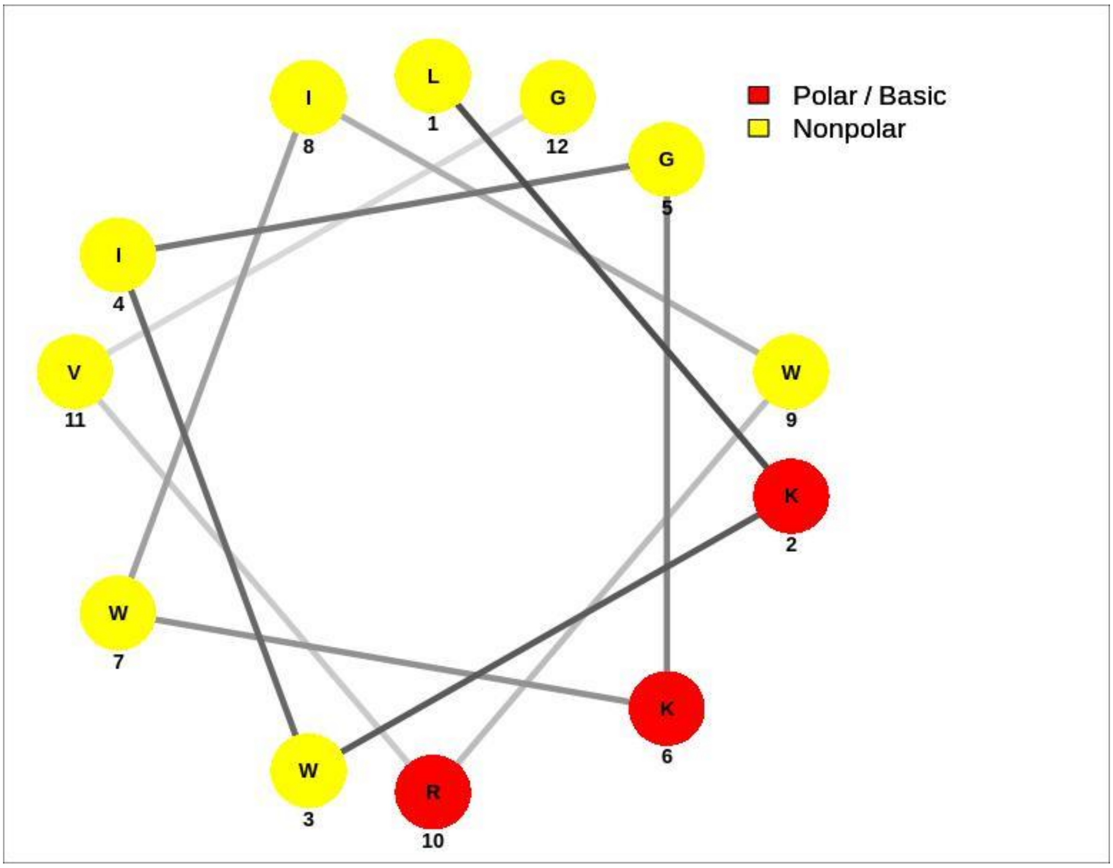
L
1
G
12
G
5
W
9
K
2
K
6
R
10
W
3
W
7
V
11
I
4
I
8
Polar / Basic
Nonpolar

C10

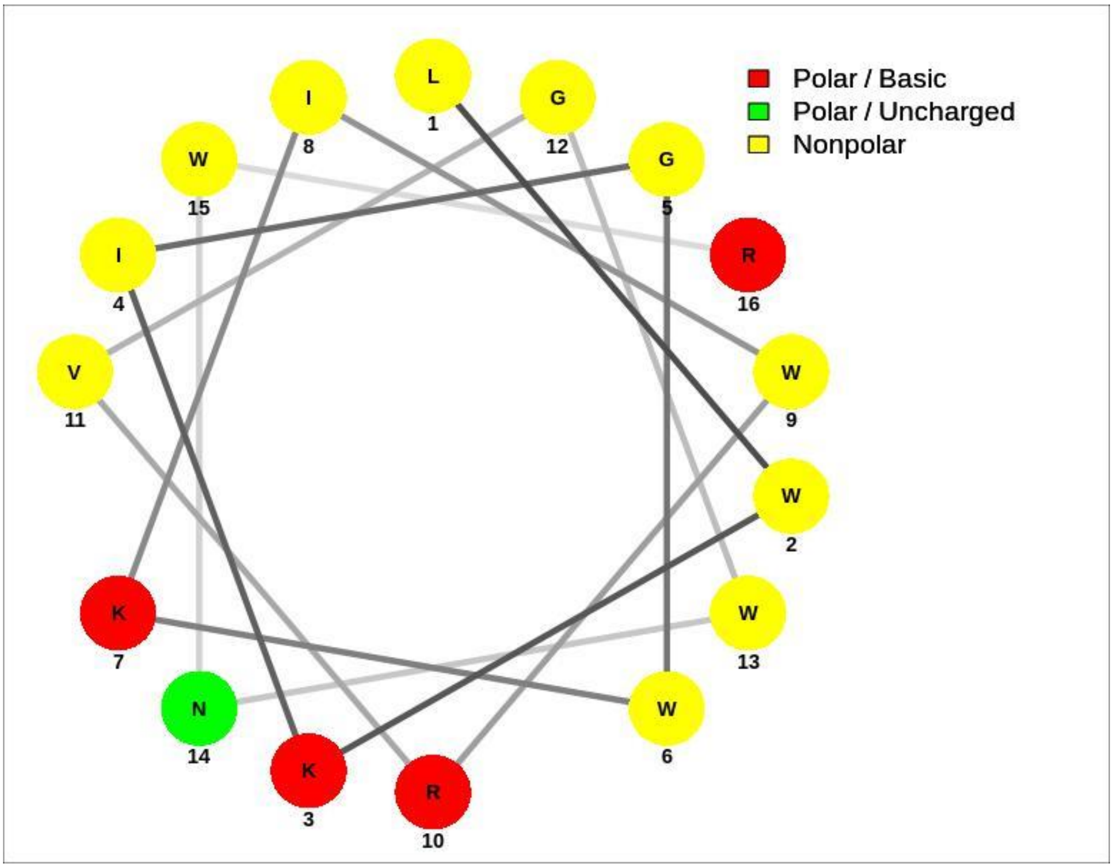
Polar / Basic
Polar / Uncharged
Nonpolar
L 1
G 12
G 5
R 16
W 9
W 2
W 13
W 6
R 10
K 3
N 14
K 7
V 11
I 4
W 15
I 8

C11

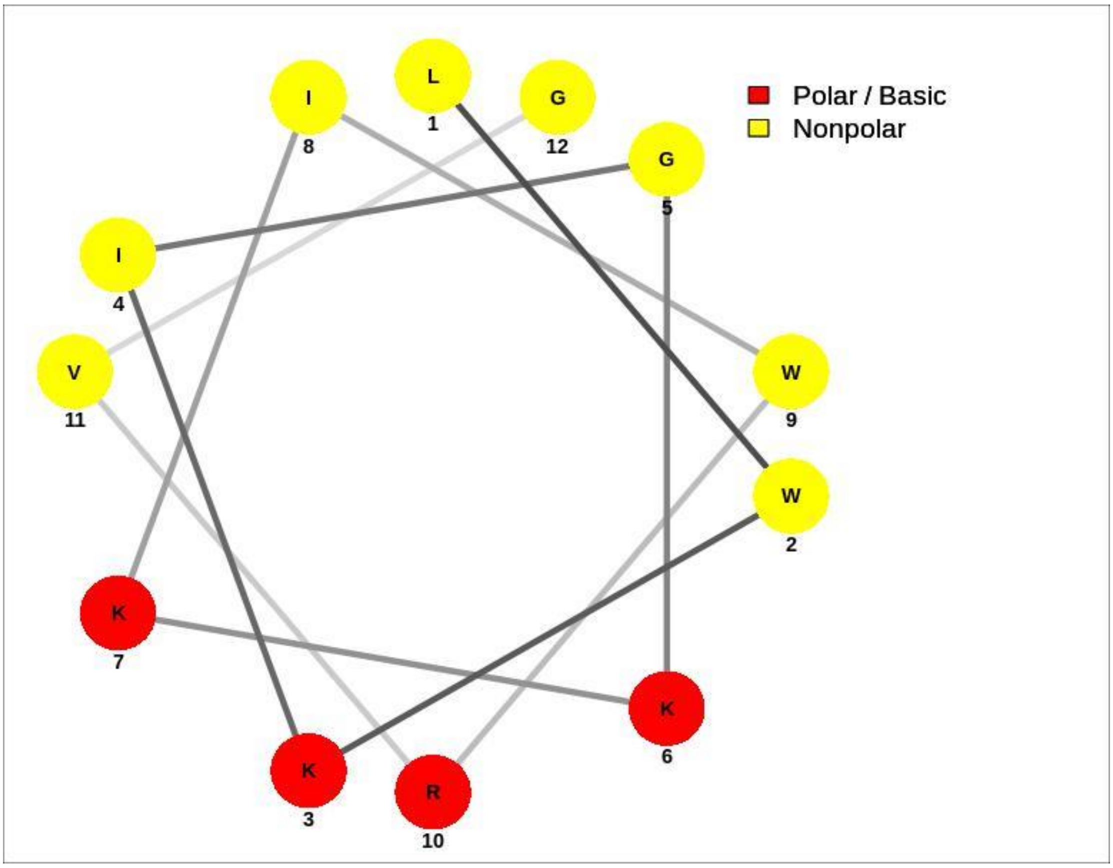
Polar / Basic
Nonpolar
L 1
G 12
G 5
W 9
W 2
K 6
R 10
K 3
K 7
V 11
I 4
I 8

C12

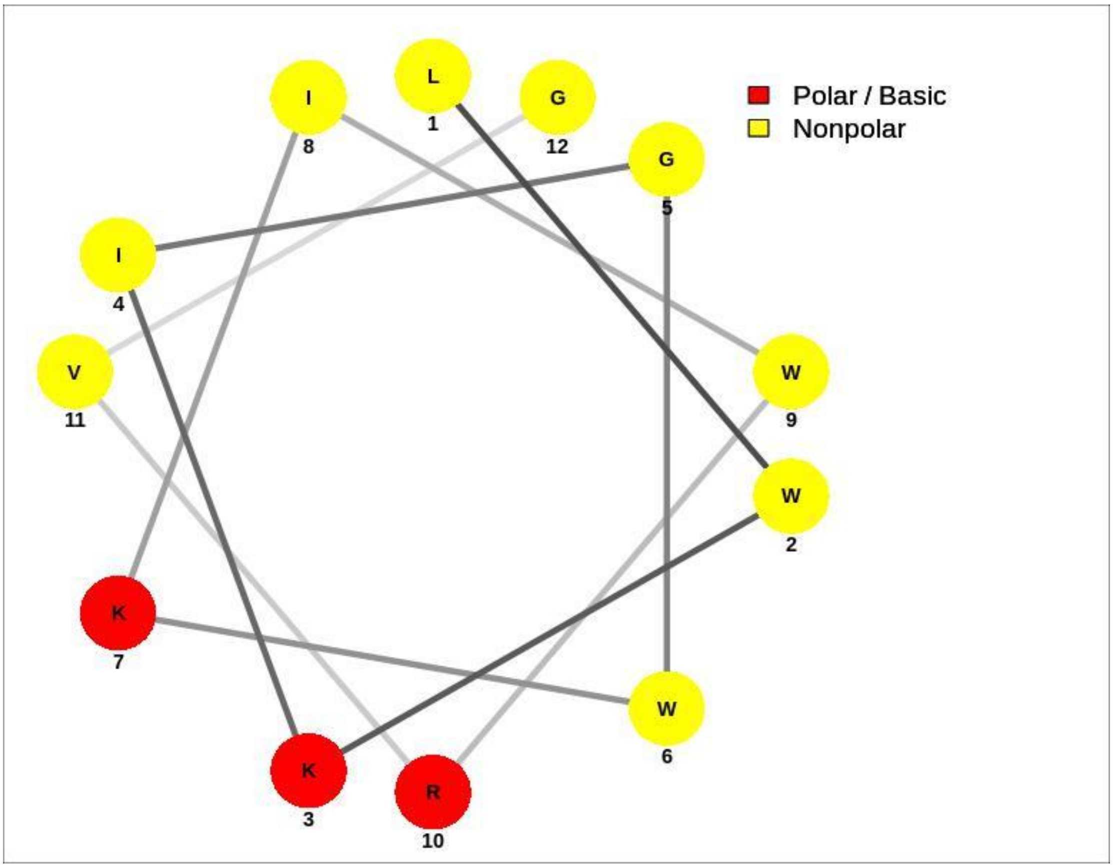

Polar / Basic
Nonpolar
L
1
W
2
K
3
I
4
G
5
W
6
K
7
I
8
W
9
R
10
V
11
G
12

C13

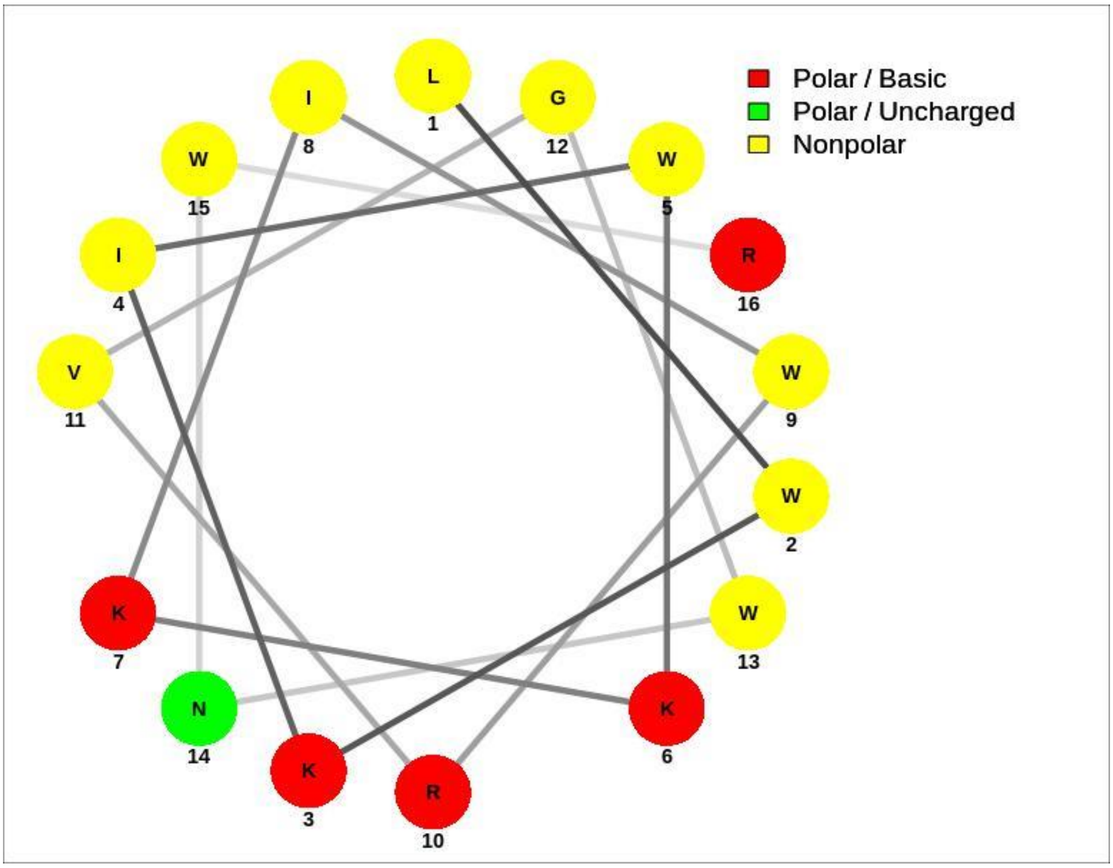

Polar / Basic
Polar / Uncharged
Nonpolar
L
1
W
2
K
3
I
4
W
5
K
6
K
7
I
8
W
9
R
10
V
11
G
12
W
13
N
14
W
15
R
16

C14

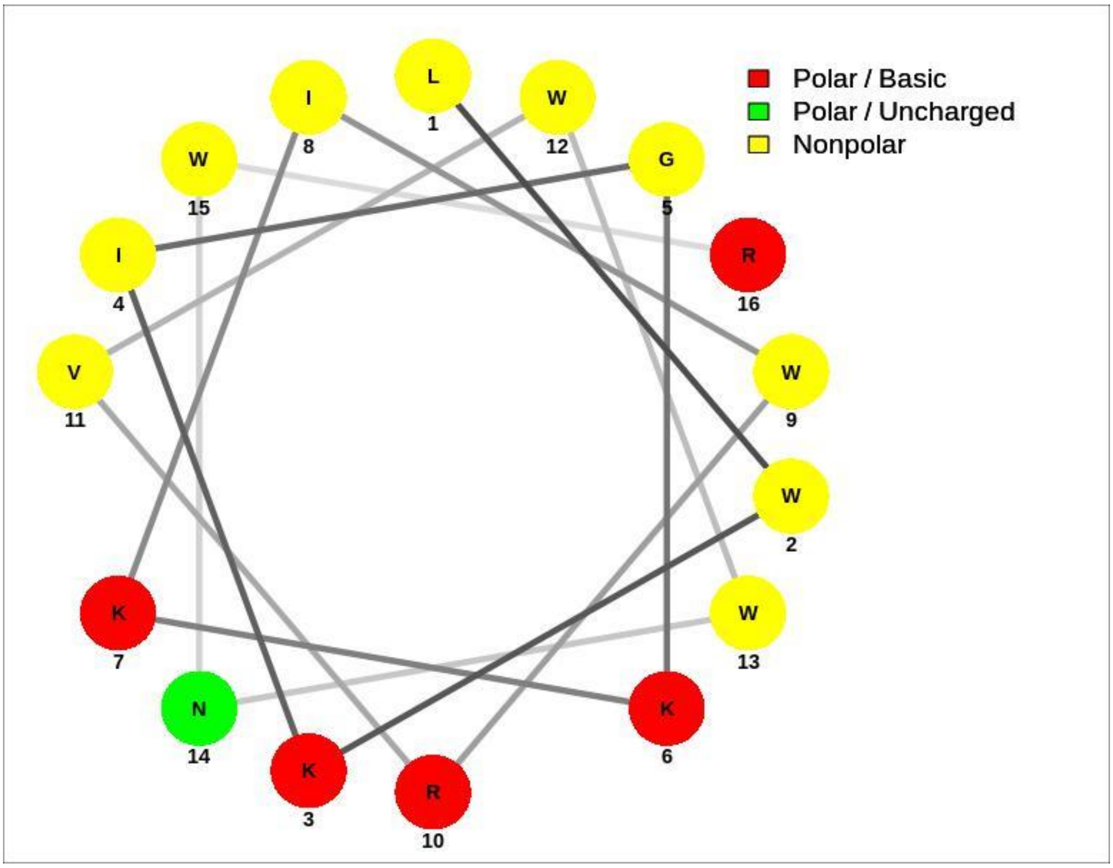
Polar / Basic
Polar / Uncharged
Nonpolar
L 1
W 2
K 3
I 4
G 5
K 6
K 7
I 8
W 9
R 10
V 11
W 12
W 13
N 14
W 15
R 16

C15

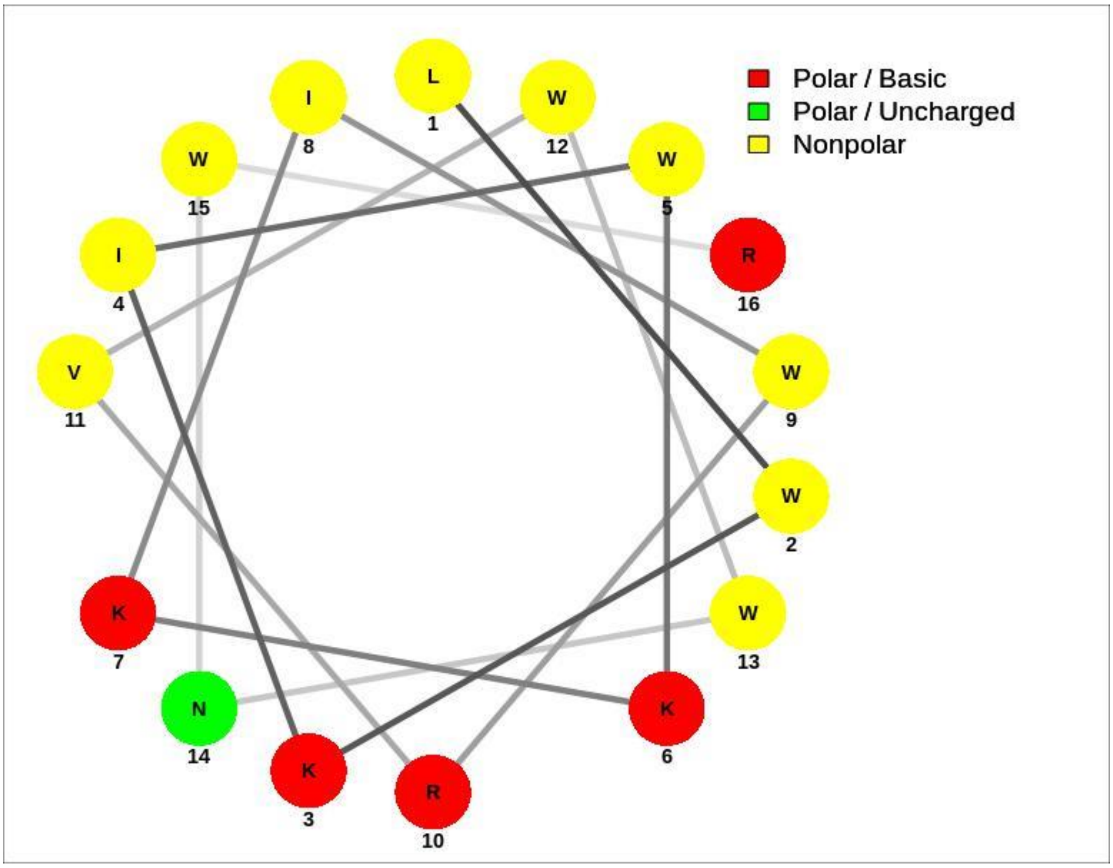
Polar / Basic
Polar / Uncharged
Nonpolar
L 1
W 2
K 3
I 4
W 5
K 6
K 7
I 8
W 9
R 10
V 11
W 12
W 13
N 14
W 15
R 16

C16

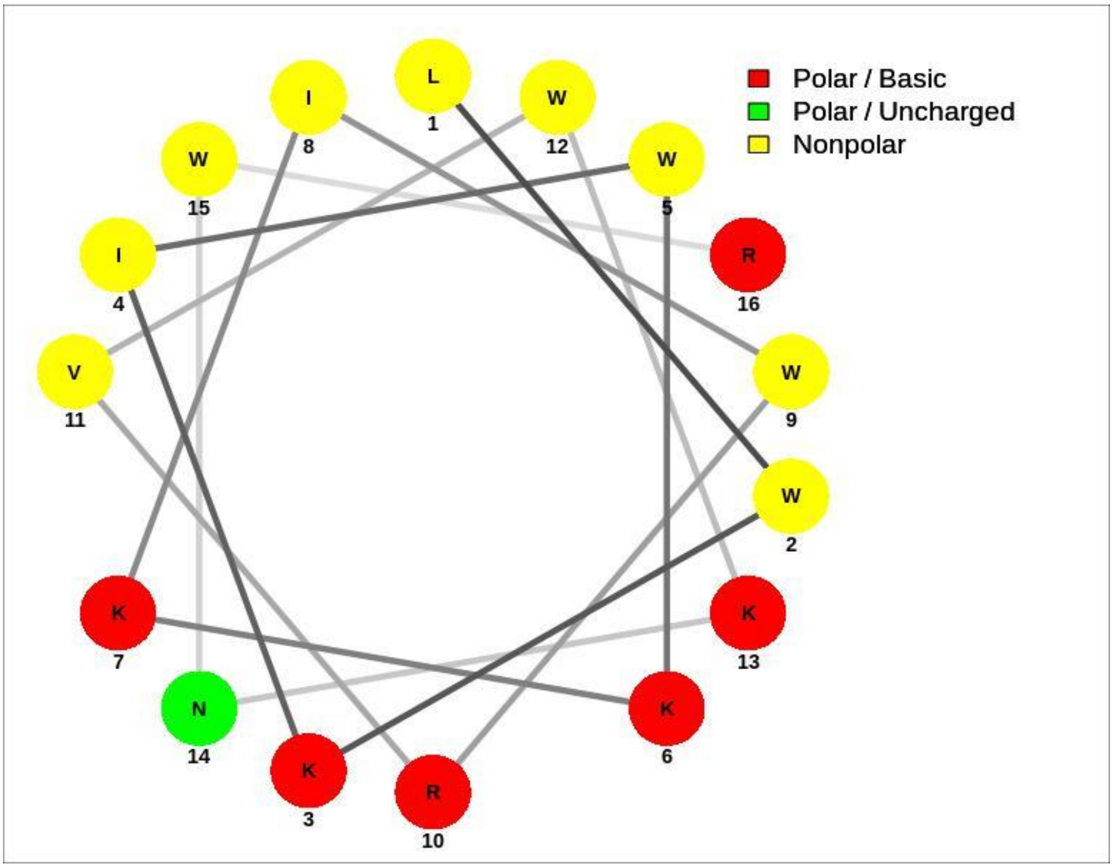
Polar / Basic
Polar / Uncharged
Nonpolar
L 1
W 2
K 3
I 4
W 5
K 6
K 7
I 8
W 9
R 10
V 11
W 12
K 13
N 14
W 15
R 16

C17

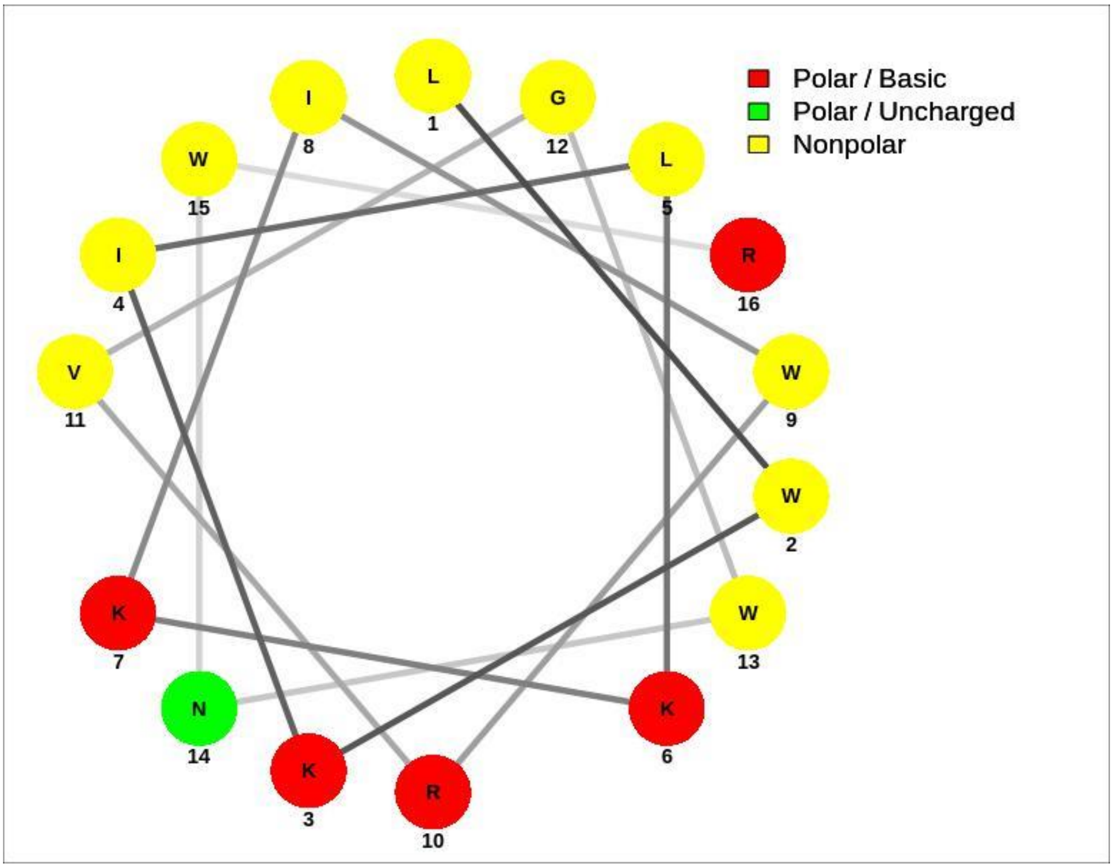
Polar / Basic
Polar / Uncharged
Nonpolar
L 1
W 2
K 3
I 4
L 5
K 6
K 7
I 8
W 9
R 10
V 11
G 12
W 13
N 14
W 15
R 16

C19

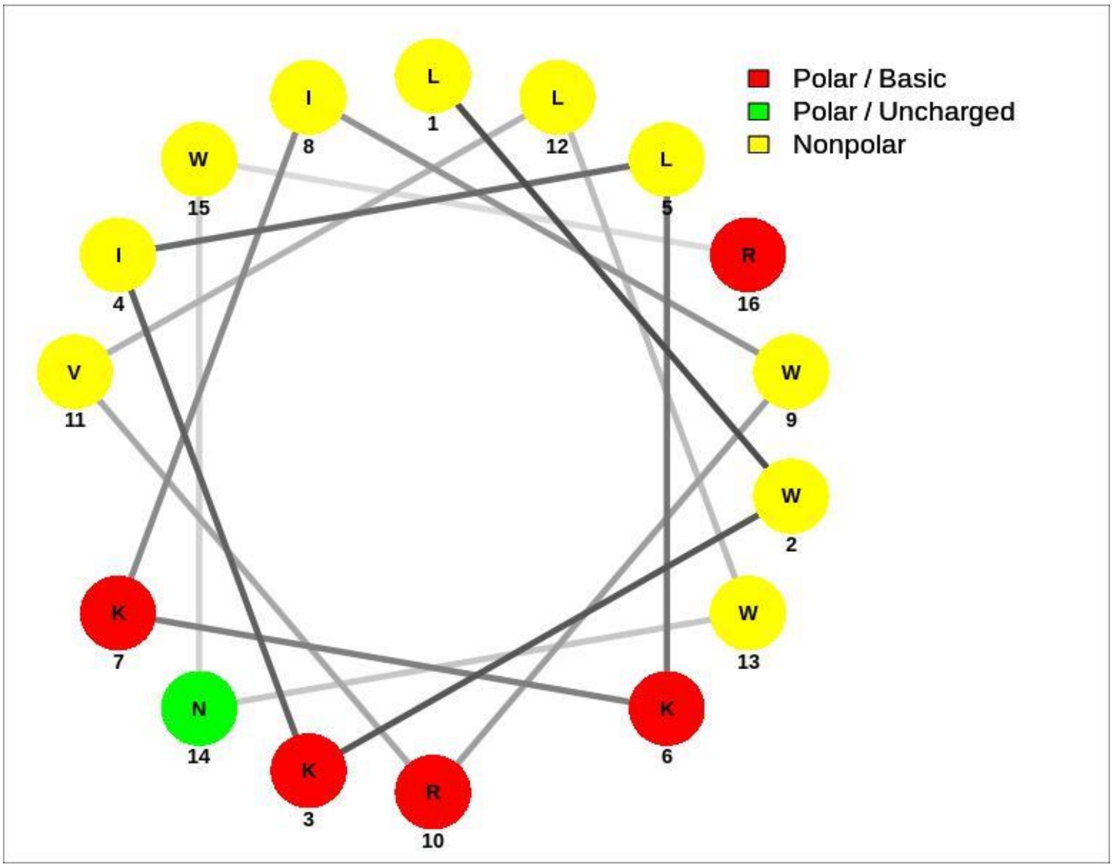
Polar / Basic
Polar / Uncharged
Nonpolar
L 1
W 2
K 3
I 4
L 5
K 6
K 7
I 8
W 9
R 10
V 11
L 12
W 13
N 14
W 15
R 16

C20

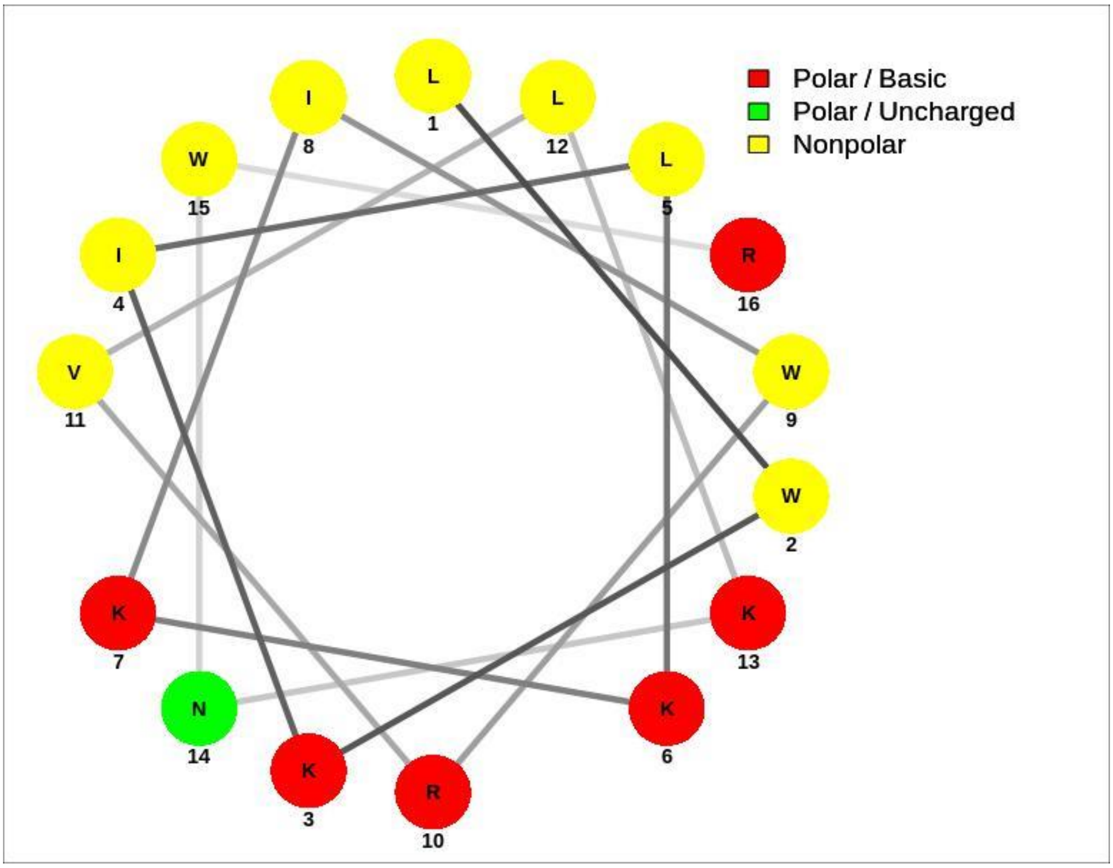
Polar / Basic
Polar / Uncharged
Nonpolar
L 1
W 2
K 3
I 4
L 5
K 6
K 7
I 8
W 9
R 10
V 11
L 12
K 13
N 14
W 15
R 16

C21

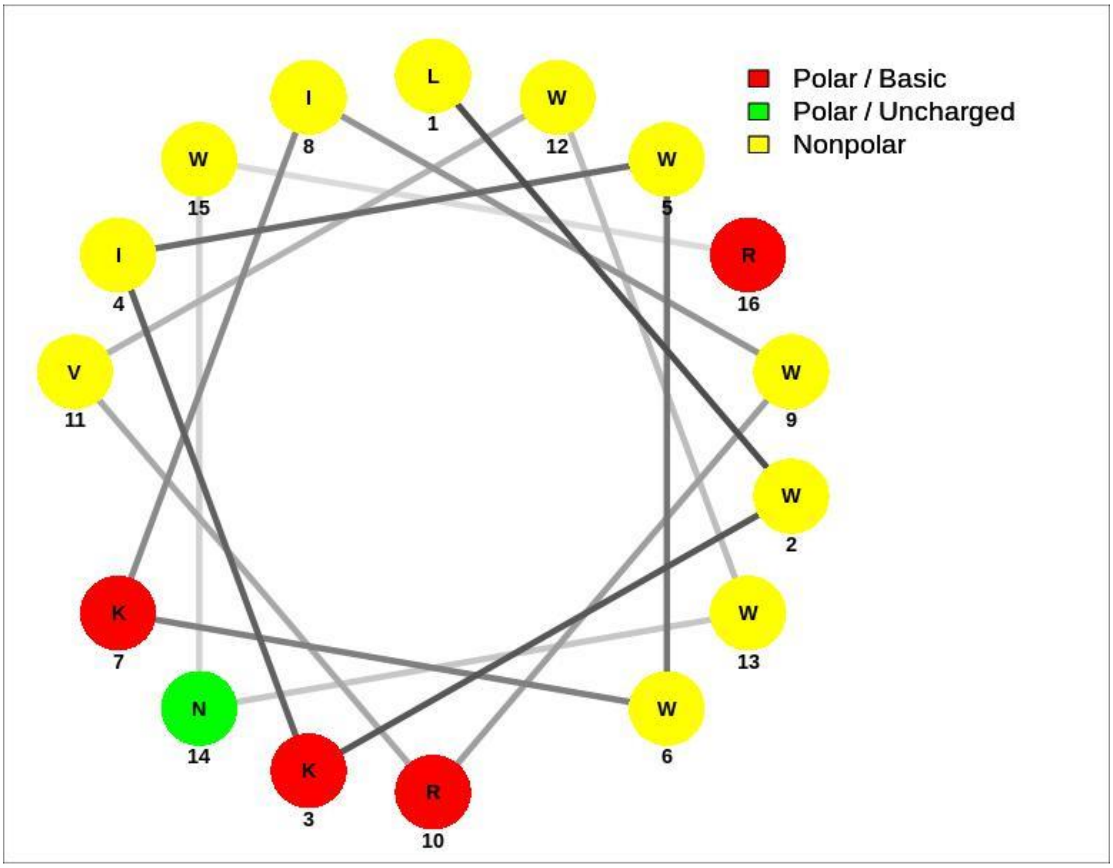

Polar / Basic
Polar / Uncharged
Nonpolar
L 1
W 12
W 5
R 16
W 9
W 2
W 13
W 6
R 10
K 3
N 14
K 7
V 11
I 4
W 15
I 8

C22

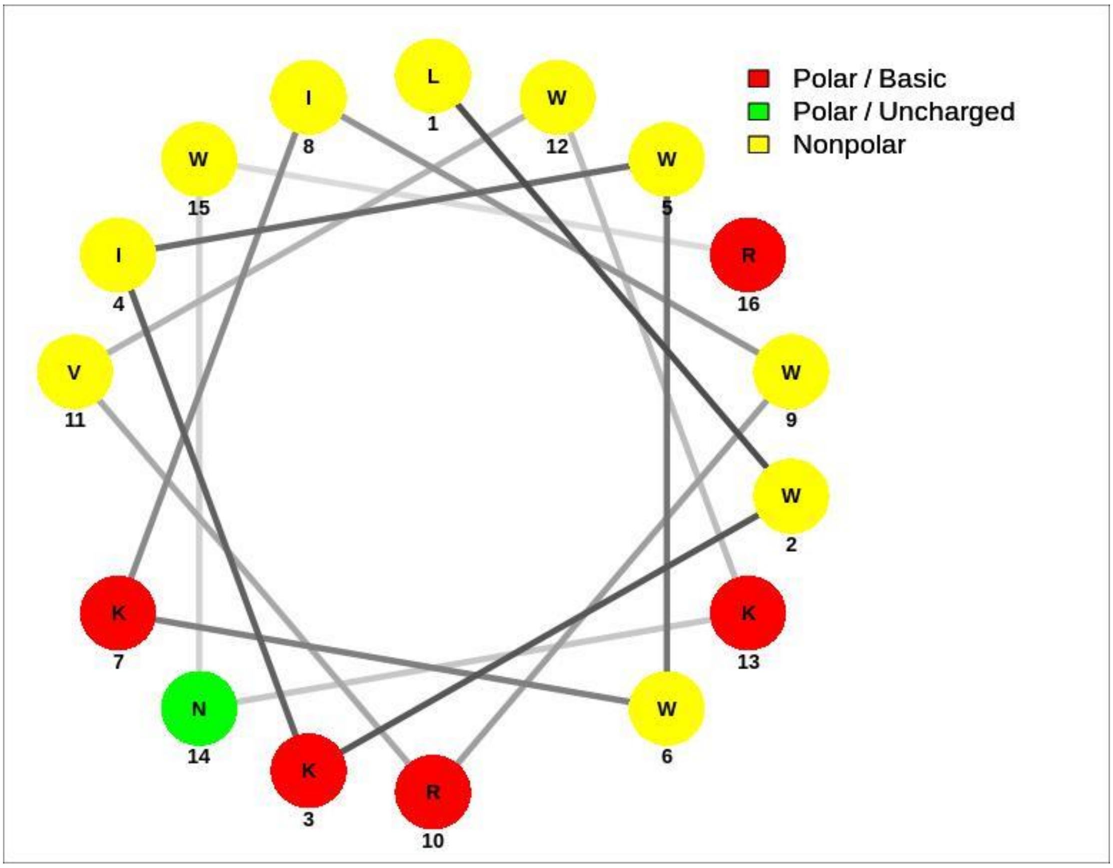

Polar / Basic
Polar / Uncharged
Nonpolar
L 1
W 12
W 5
R 16
W 9
W 2
K 13
W 6
R 10
K 3
N 14
K 7
V 11
I 4
W 15
I 8

C23

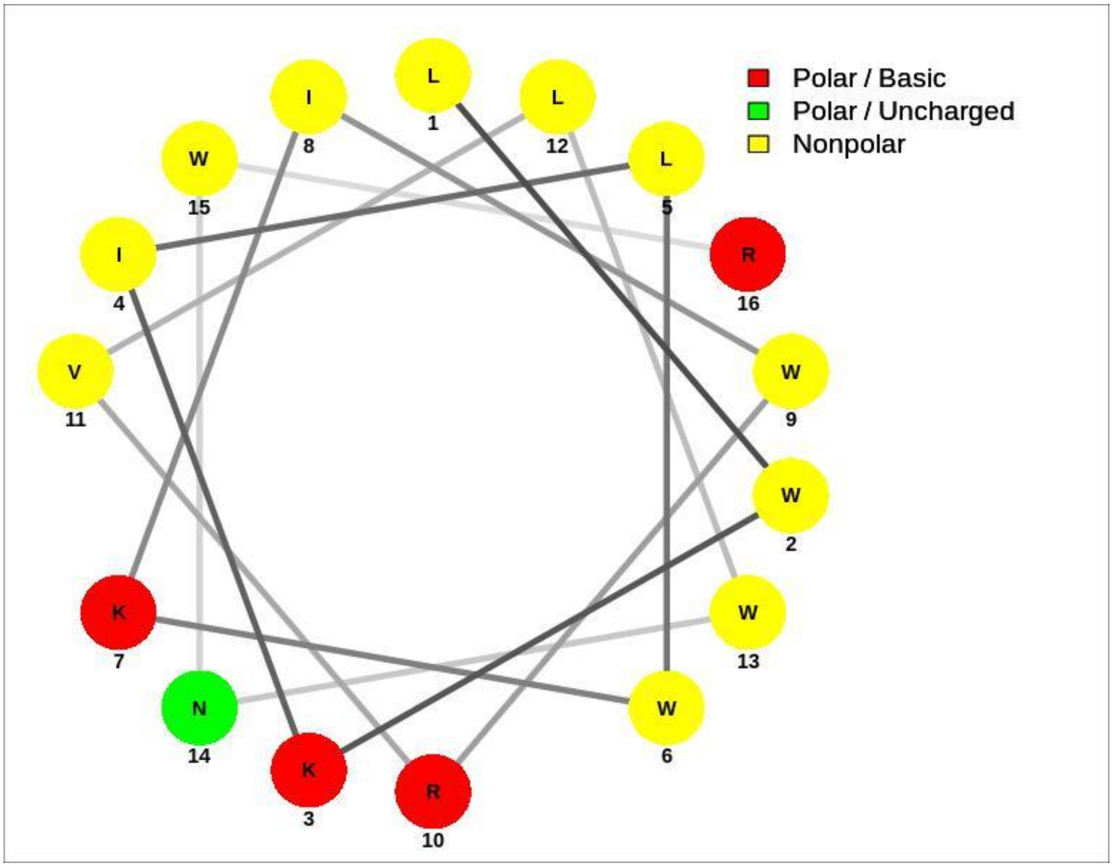
L
1
W
2
K
3
I
4
L
5
W
6
K
7
I
8
W
9
R
10
V
11
L
12
W
13
N
14
W
15
R
16
Polar / Basic
Polar / Uncharged
Nonpolar

C24

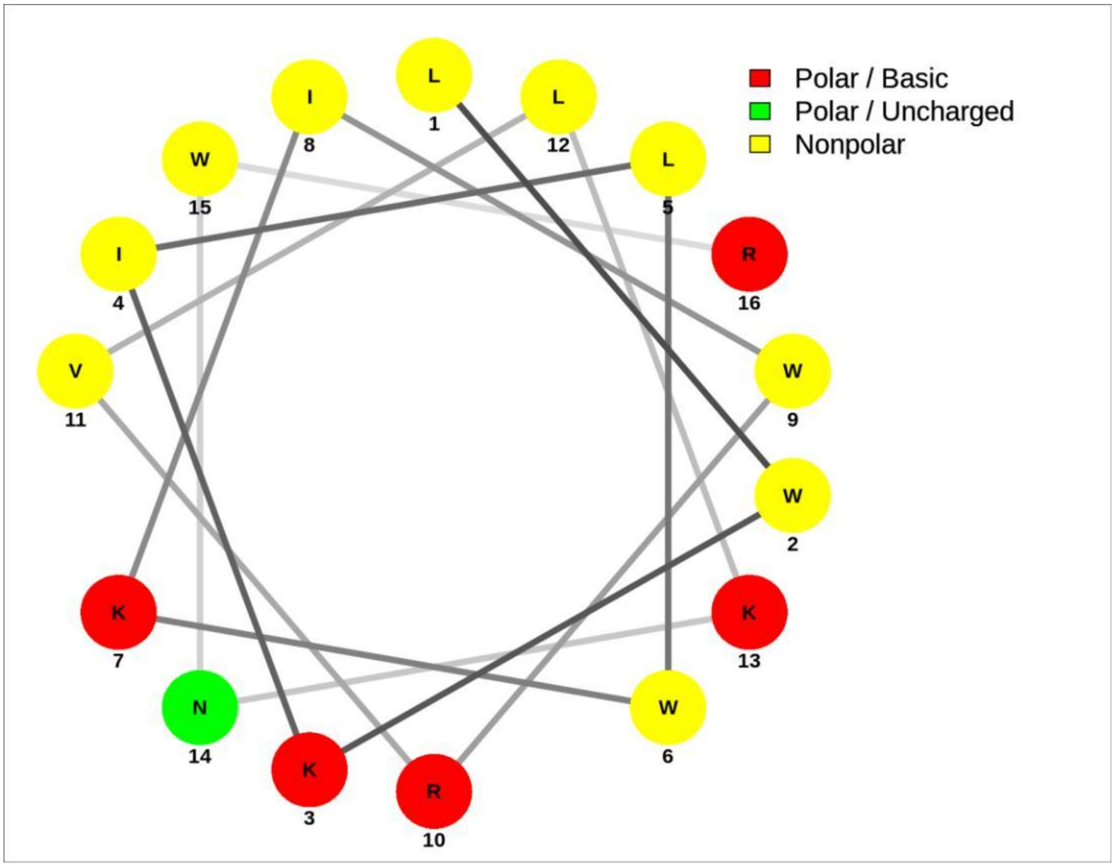
L
1
W
2
K
3
I
4
L
5
W
6
K
7
I
8
W
9
R
10
V
11
L
12
K
13
N
14
W
15
R
16
Polar / Basic
Polar / Uncharged
Nonpolar

Figure S2. The MS and HPLC information of synthetic peptides

MASS SPECTROMETRY REPORT -C1

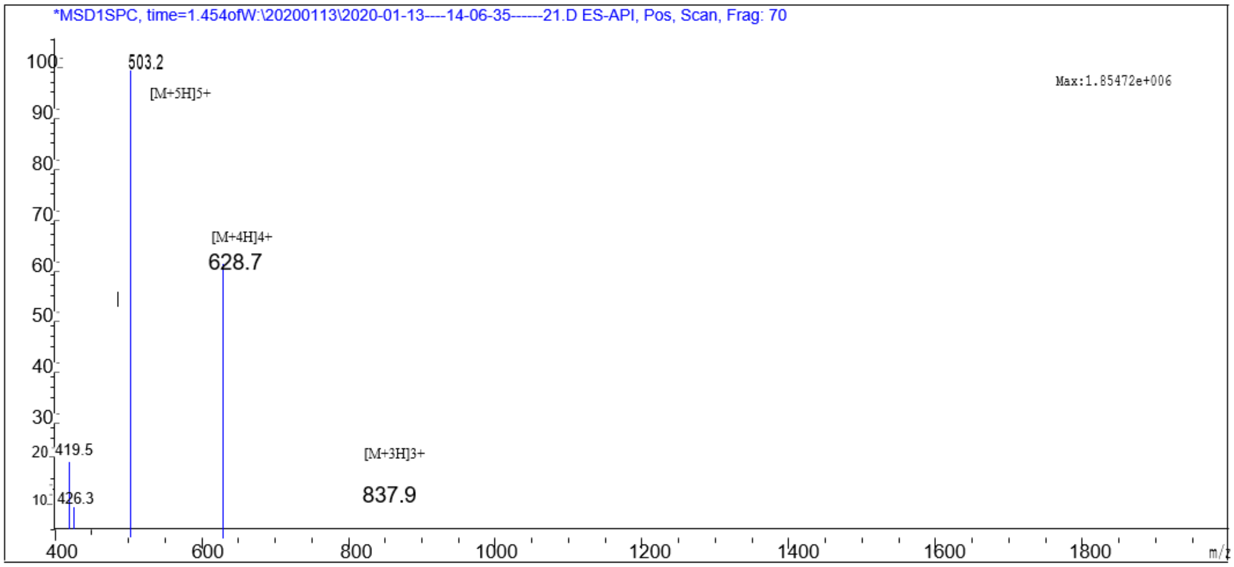

Sample Description		Instrument	Agilent-6125B		
Analyzed date:	2020-01-13	Probe:	ESI	Probe Bias:	+ 4.5kv
Analyst:	YU	Nebulizer Gas Flow:	1.5L/min	Detector:	1.5kv
Sample:	C1 LQ-21	CDL:	-20.0v	T. Flow:	0.2ml/min
M.W.:	2511.04	CDL Temp.:	250 ºC	B. Conc.:	50%H2O/50%ACN
Lot. No.:	P200106-MJ772750	Block Temp.:	200 ºC		

HPLC REPORT -C1

Product Name:C1 LQ-21
Instrument No: 0200194
Lot No :P200106-MJ772750
Column :4.6*250mm C18
Solvent A :0.1%Trifluoroacetic in 100% Acetonitrile
Solvent B :0.1%Trifluoroacetic in 100% Water

Gradient :	A	B
0.01min	20%	80%
25min	45%	55%
25.01min	100%	0%
30.0min	STOP	

Flow rate :1.0ml/min
Wavelength :220nm
Volume: 10μl

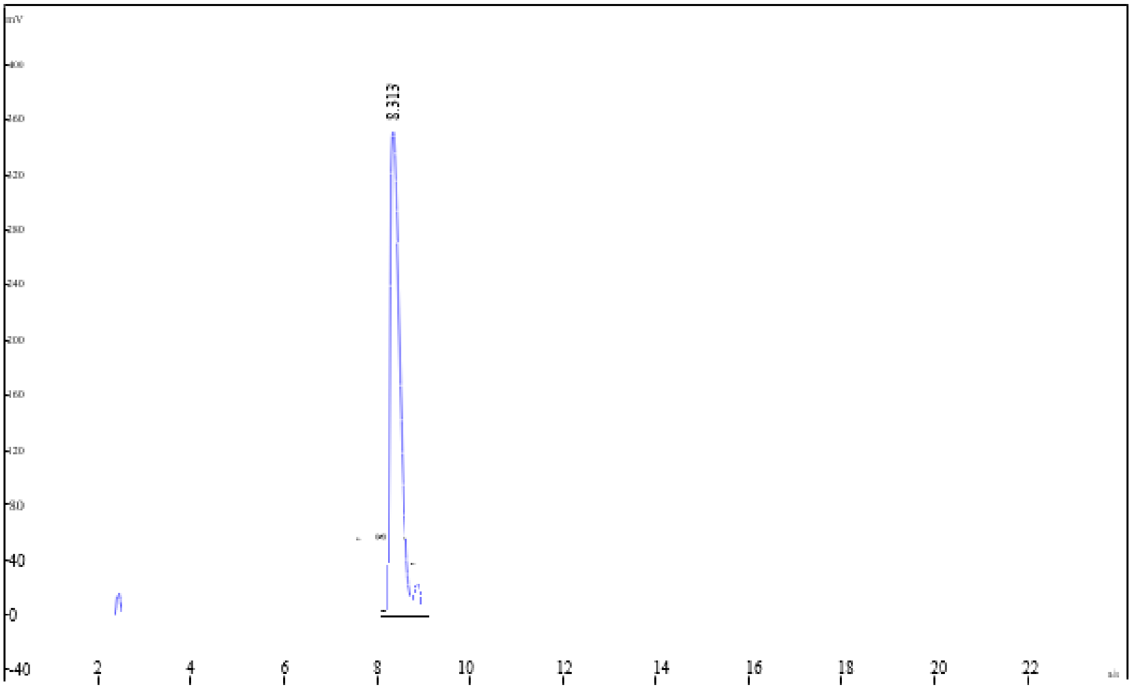

Rank	Time	Name	Conc.	Area	Height
1	8.205		0.07226	3340	2492
2	8.313		95.98	4436853	348374
3	8.849		3.946	182381	19720
Total			100	4622574	370586

MASS SPECTROMETRY REPORT -C2

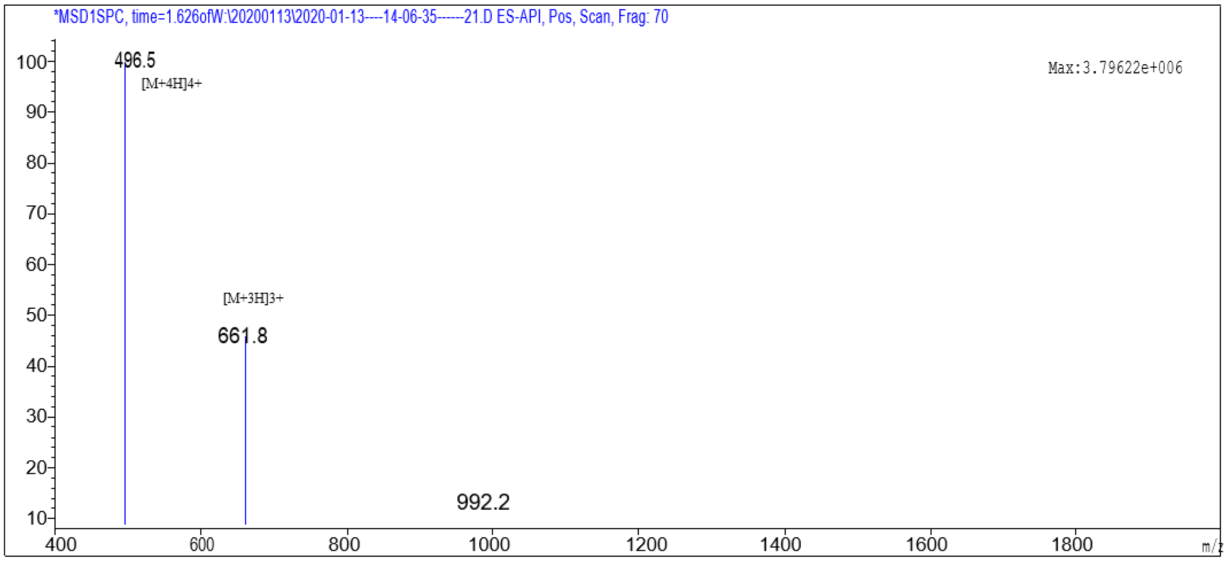

Sample Description		Instrument	Agilent-6125B		
Analyzed date:	2020-01-13	Probe:	ESI	Probe Bias:	+ 4.5kv
Analyst:	YU	Nebulizer Gas Flow:	1.5L/min	Detector:	1.5kv
Sample:	C2 LR-16	CDL:	-20.0v	T. Flow:	0.2ml/min
M.W.:	1982.47	CDL Temp.:	250 ºC	B. Conc.:	50%H2O/50%ACN
Lot. No.:	P200106-MJ772751	Block Temp.:	200 ºC		

HPLC REPORT −C2

Product Name:C2 LR-16
Instrument No: 0200194
Lot No :P200106-MJ772751
Column :4.6*250mm C18
Solvent A :0.1%Trifluoroacetic in 100% Acetonitrile
Solvent B :0.1%Trifluoroacetic in 100% Water

Gradient :	A	B
0.01min	16%	84%
25min	41%	59%
25.01min	100%	0%
30.0min	STOP	

Flow rate :1.0ml/min
Wavelength :220nm
Volume: 10μl

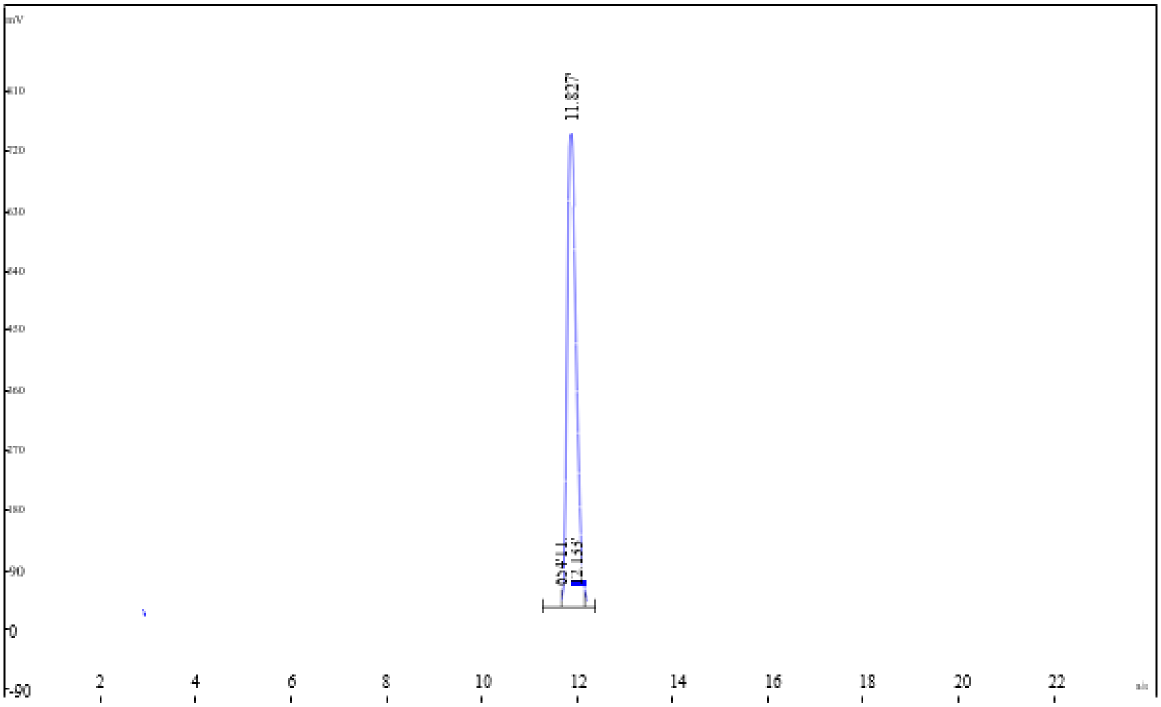

Rank	Time	Name	Conc.	Area	Height
1	11.654		1.683	148100	14151
2	11.827		97.84	8609738	711574
3	12.133		0.4731	41628	13487
Total			100	8799466	739212

MASS SPECTROMETRY REPORT -C3

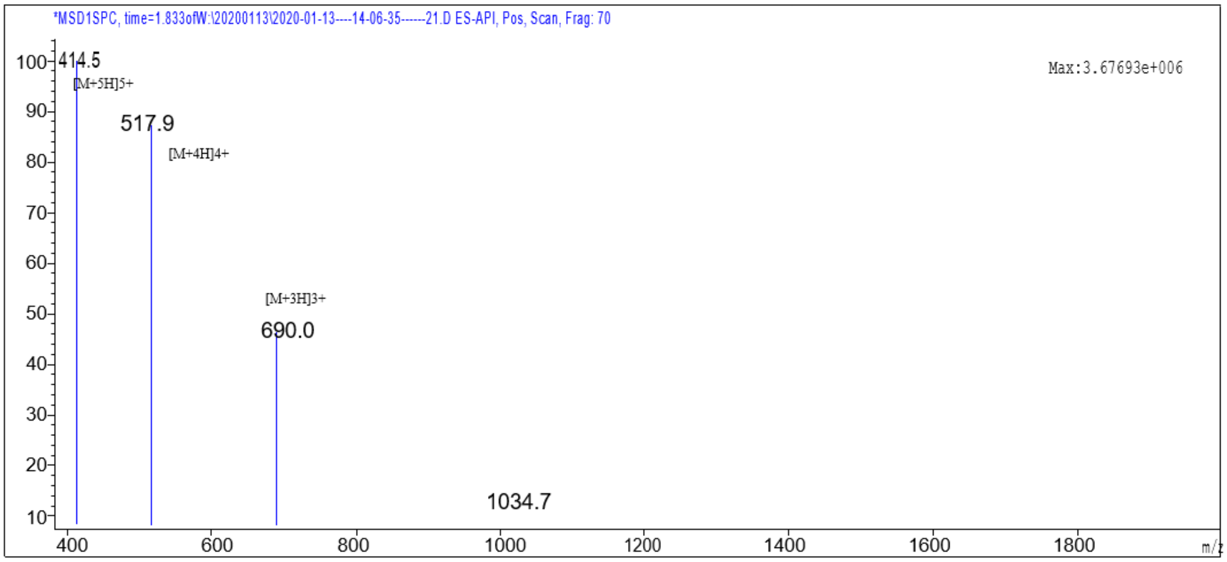

Sample Description		Instrument	Agilent-6125B		
Analyzed date:	2020-01-13	Probe:	ESI	Probe Bias:	+4.5kv
Analyst:	YU	Nebulizer Gas Flow:	1.5L/min	Detector:	1.5kv
Sample:	C3 LR-16	CDL:	-20.0v	T. Flow:	0.2ml/min
M.W.:	2067.58	CDL Temp.:	250 ºC	B. Conc.:	50%H2O/50%ACN
Lot. No.:	P200106-MJ772752	Block Temp.:	200 ºC		

HPLC REPORT -C3

Product Name:C3 LR-16
Instrument No: 0200194
Lot No :P200106-MJ772752
Column :4.6*250mm C18
Solvent A :0.1%Trifluoroacetic in 100% Acetonitrile
Solvent B :0.1%Trifluoroacetic in 100% Water

Gradient :	A	B
0.01min	21%	79%
25min	46%	54%
25.01min	100%	0%
30.0min	STOP	

Flow rate :1.0ml/min
Wavelength :220nm
Volume: 10μl

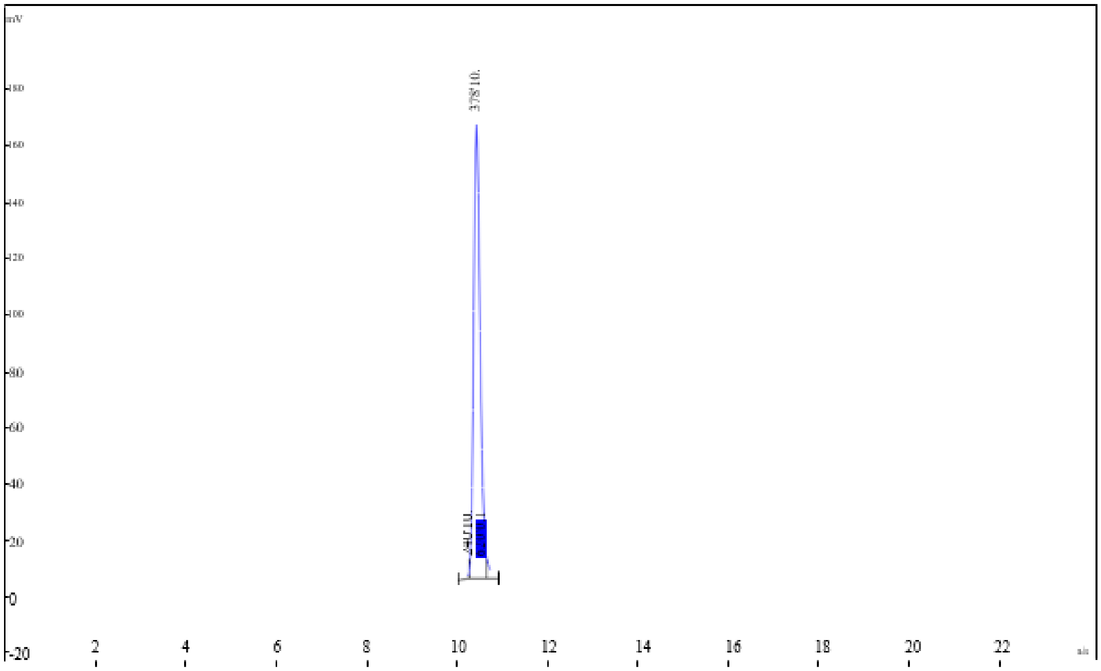

Rank	Time	Name	Conc.	Area	Height
1	10.240		0.6636	10208	4521
2	10.378		97.75	1503781	161147
3	10.620		1.584	24374	5448
Total			100	1538363	171116

Mass Spectrum -C4

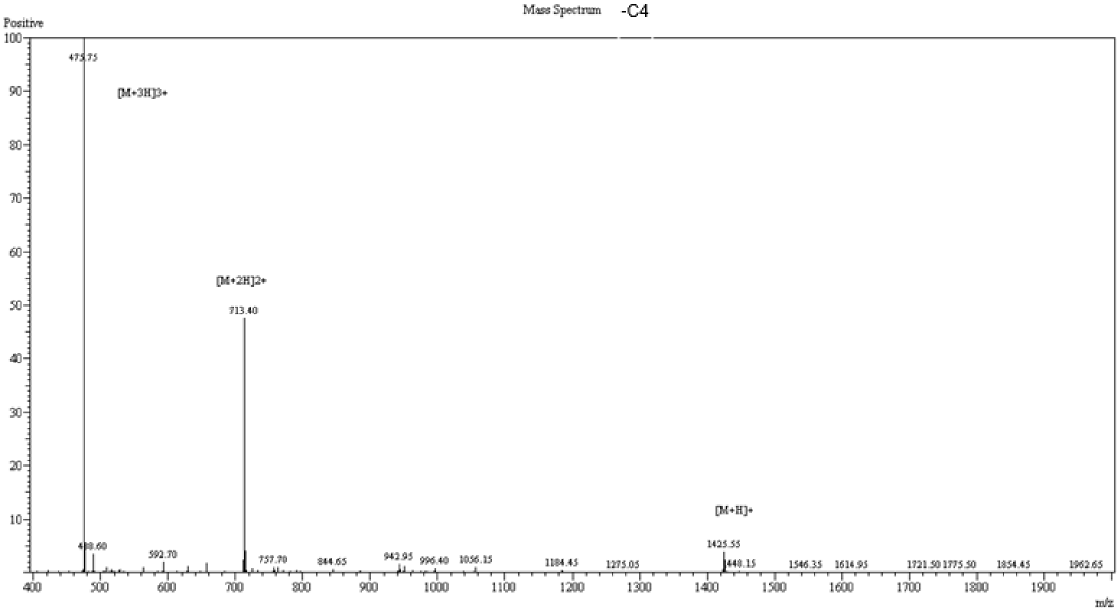

Sample Information		Probe:	ESI	Probe bias:	-3.5kv
Date and Time	: 2020-1-10 10:17:28	Nebulizer Gas Flow:	1.5L/min	Detector:	1.0kv
User	: CHAO	DL:	-20.0v	T. Flow:	0.2ml/min
Sample	: CT4 LG-12	DL Temp:	250℃	B. conc:	50%H2O/50%ACN
Inj. Volume	: 1	Block Temp:	200℃		
MW	:1424.86				
Lot No.	:P200106-MJ772753				

HPLC -C4

Sample Description:
Analyst:YSC
Structure:C4 LG-12
Lot NO.:P200106-MJ772753
Number:0200193
Column:250*4.6mm Boston Green 0DS-AQ
Solvent A:0.1%TFA in 100%water
Solvent B:0.1%TFA in 100%acetonitrile

Gradient :	A	B
0.1min	88	12
25min	63	37
25.01min	0	100
30min	0	100

Flow rate:1.0ml/min
Wavelength(nm):220
Volume:10ul
File opened: D:\2020 HPLC\lg-12-f 772753 200113.hw, where

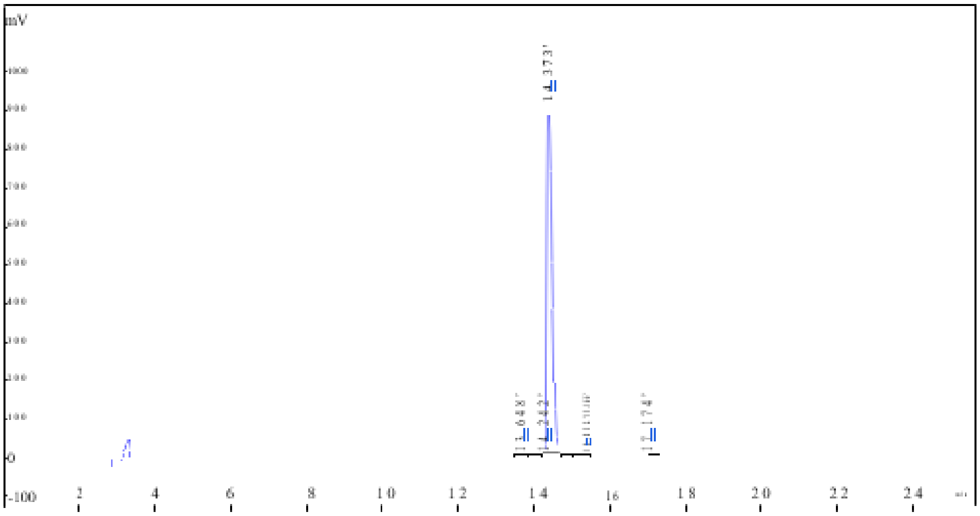

Rank	Time	Conc.	Area	Height
1	13.648	0.4177	35391	5086
2	14.242	0.3492	29584	3645
3	14.373	97.6	8269514	873551
4	14.832	0.5624	47647	4250
5	15.166	0.8562	72540	8631
6	17.174	0.2126	18009	2708
Total		100	8472685	897871

Mass Spectrum -C5

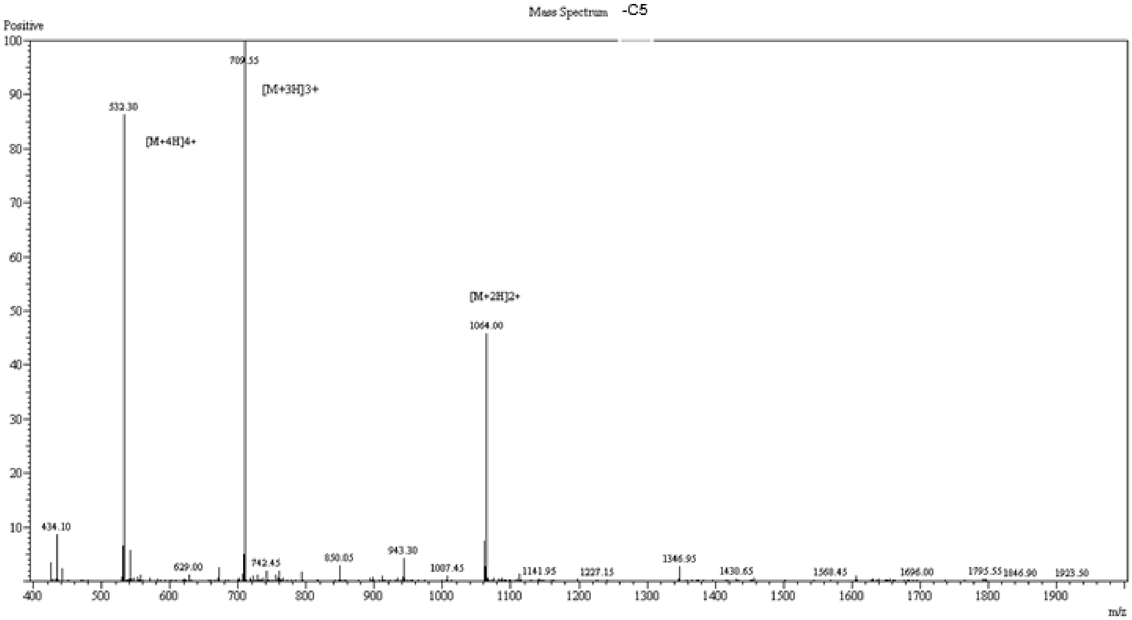

Sample Information	
Date and Time	: 2020-1-10 10:17:28
User	: CHAO
Sample	: CT5 LR-16
Inj. Volume	: 1
MW	:2125.62
Lot No.	:P200106-MJ772754

Probe:	ESI	Probe bias:	-3.5kv
Nebulizer Gas Flow:	1.5L/min	Detector:	1.0kv
DL:	-20.0v	T. Flow:	0.2ml/min
DL Temp:	250℃	B. conc:	50%H2O/50%ACN
Block Temp:	200℃		

HPLC -C5

Sample Description:
Analyst:YSC
Structure:C5 LR-16
Lot NO :P200106-MJ772754
Number:0200193
Column:250*4.6mm Boston Green 0DS-AQ
Solvent A:0.1%TFA in 100%water
Solvent B:0.1%TFA in 100%acetonitrile

Gradient :	A	B
0.1min	80	20
25min	55	45
25.01min	0	100
30min	0	100

Flow rate:1.0ml/min
Wavelength(nm):220
Volume:10ul
File opened: D:\2020 HPLC\LR-16-F 772754 200113.hw, where

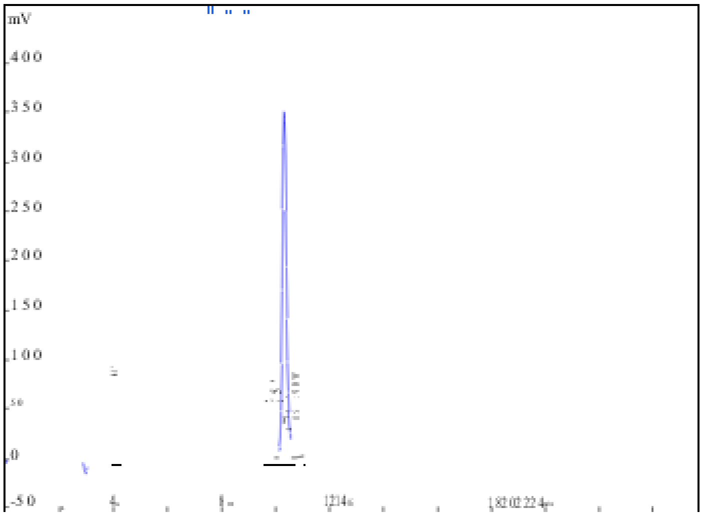

Rank	Time	Conc.	Area	Height
1	4.041	1.053	39379	4898
2	9.841	0.554	20719	2056
3	10.105	0.9643	36066	7942
4	10.279	95.15	3558855	348839
5	10.590	1.775	66385	12908
6	10.750	0.5015	18756	2746
Total		100	3740160	379389

Mass Spectrum -C6

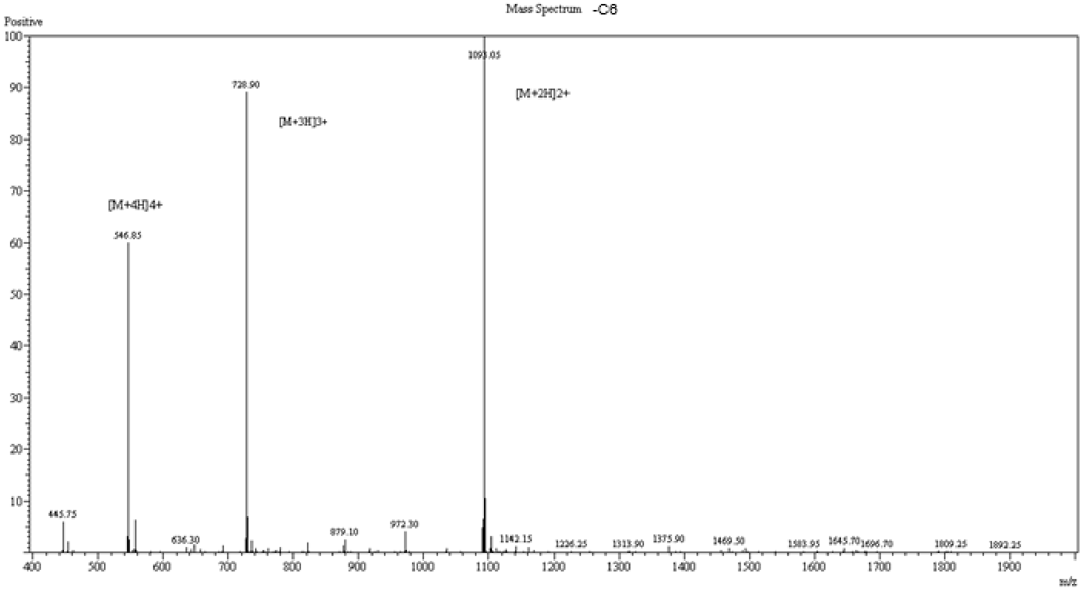

Sample Information
Date and Time : 2020-1-10 10:17:28
User : CHAO
Sample :CT6 LR-16
Inj. Volume : 1
MW :2183.66
Lot No. :P200106-MJ772755

Probe: ESI Probe bias: -3.5kv
Nebulizer Gas Flow: 1.5L/min Detector: 1.0kv
DL: -20.0v T. Flow: 0.2ml/min
DL Temp: 250℃ B. conc: 50%H2O/50%ACN
Block Temp: 200℃

HPLC -C6

Sample Description:
Analyst:YSC
Structure:C6 LR-16
Lot NO :P200106-MJ772755
Number:0200193
Column:250*4.6mm Boston Green 0DS-AQ
Solvent A:0.1%TFA in 100%water
Solvent B:0.1%TFA in 100%acetonitrile

Gradient :	A	B
0.1min	75	25
25min	50	50
25.01min	0	100
30min	0	100

Flow rate:1.0ml/min
Wavelength(nm):220
Volume:10ul
File opened: D:\2020 HPLC\LR-16-F 772755 200113.hw, where

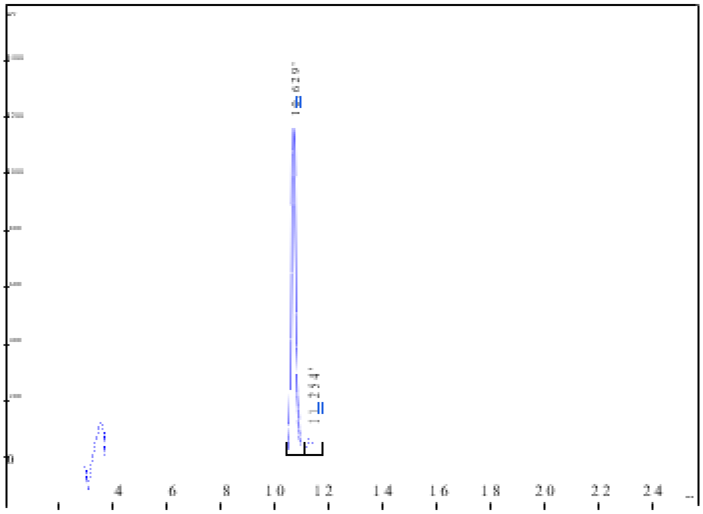

Rank	Time	Conc.	Area	Height
1	10.629	95.19	12285170	1135126
2	11.254	4.807	620363	49419
Total		100	12905533	1184545

Mass Spectrum C7

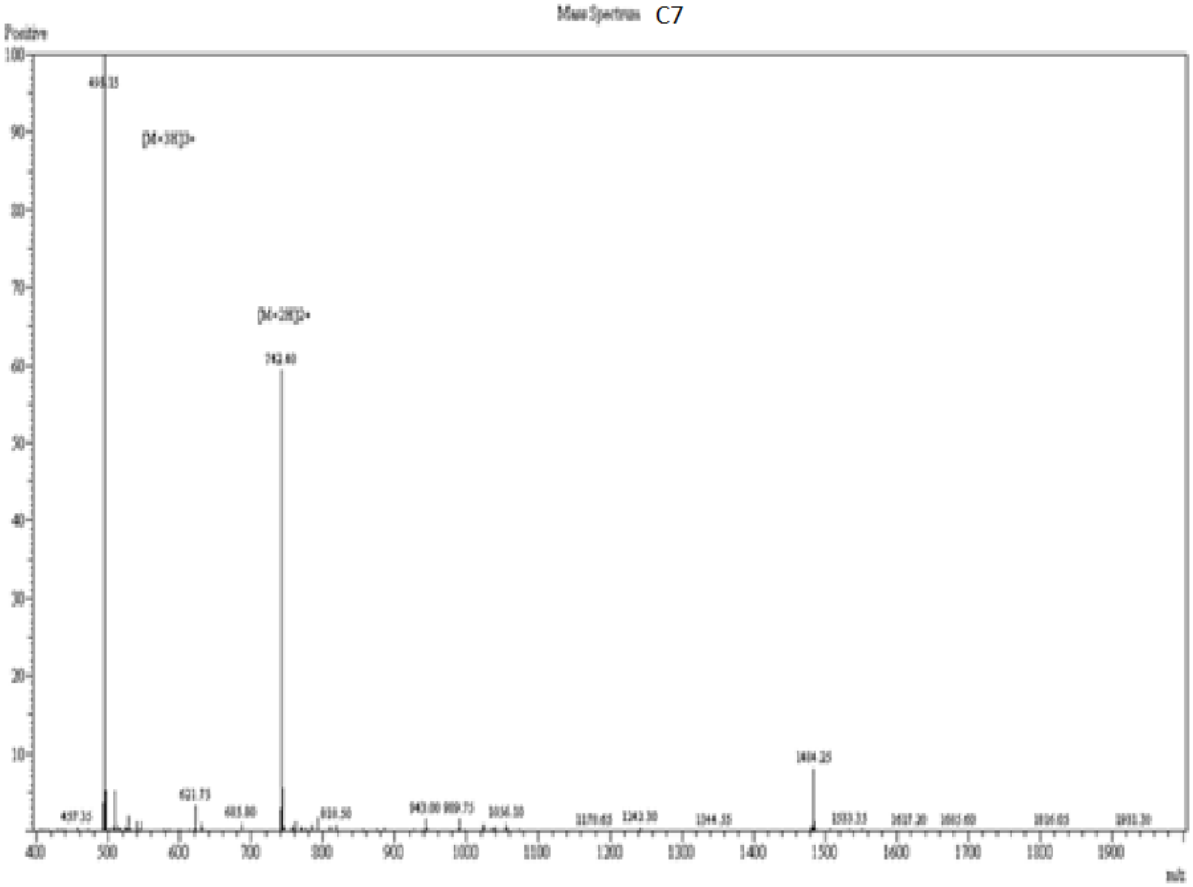

Sample Information	
Date and Time	: 2020-1-10 10:17:28
User	: CHAO
Sample	: CT7 LG-12
Inj. Volume	: 1
MW	:1482.90
Lot No.	:P200106-MJ772756

Probe:	ESI	Probe bias:	-3.5kv
Nebulizer Gas Flow:	1.5L/min	Detector:	1.0kv
DL:	-20.0v	T. Flow:	0.2ml/min
DL Temp:	250℃	B. conc:	50%H2O/50%ACN
Block Temp:	200℃		

HPLC -C7

Sample Description:
Analyst:YSC
Structure:C7 LG-12
Lot NO :P200106-MJ772756
Number:0200193
Column:250*4.6mm,Boston Green 0DS-AQ
Solvent A:0.1%TFA in 100%water
Solvent B:0.1%TFA in 100%acetonitrile

Gradient :	A	B
0.1min	82	18
25min	57	43
25.01min	0	100
30min	0	100

Flow rate:1.0ml/min
Wavelength(nm):220
Volume:10ul
File opened: D:\2020\LG-12-F 772756 200113.hw, where

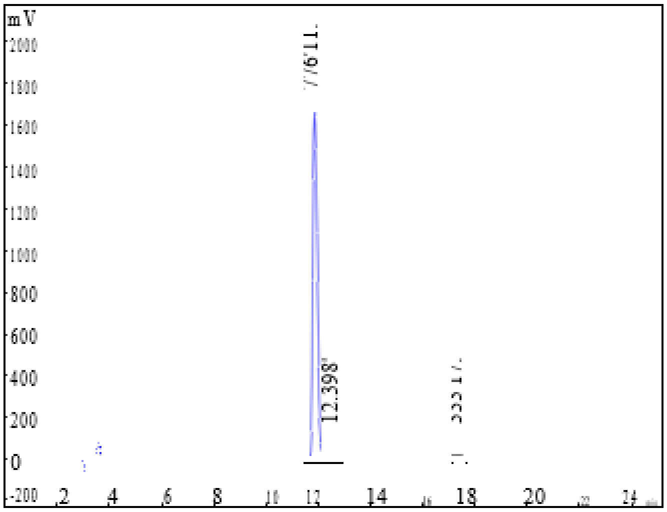

Rank	Time	Conc.	Area	Height
1	11.776	98.29	18104182	1647984
2	12.398	1.333	245526	17448
3	17.333	0.3786	69734	8281
Total		100	18419442	1673713

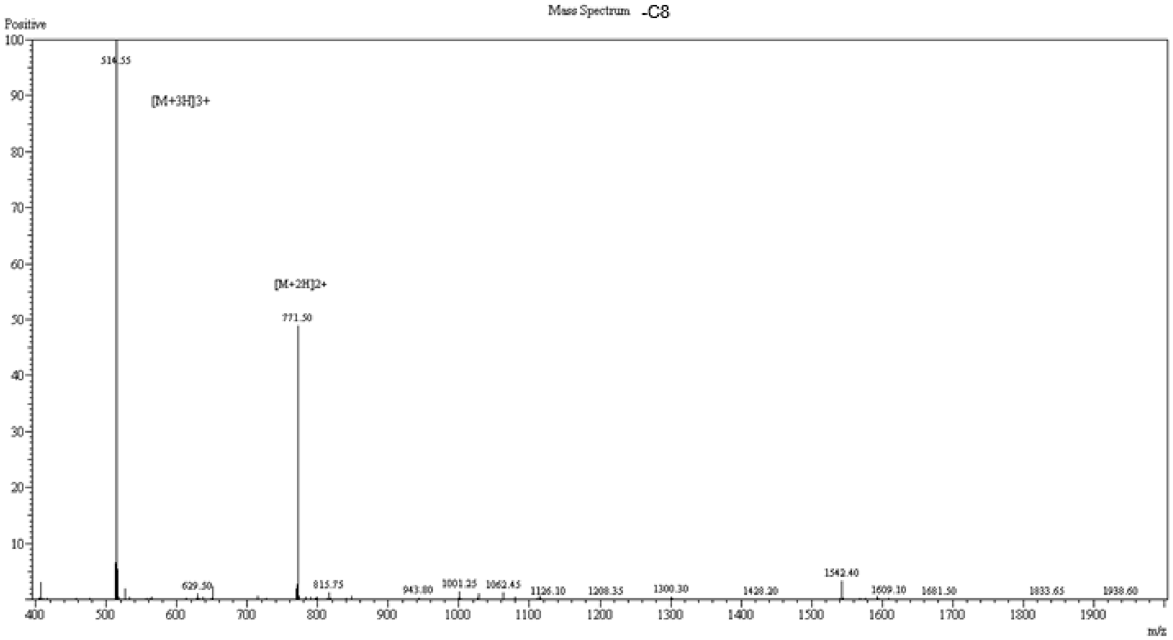

Sample Information		Probe:	ESI	Probe bias:	-3.5kv
Date and Time	: 2020-1-10 9:34:35	Nebulizer Gas Flow:	1.5L/min	Detector:	1.0kv
User	: CHAO	DL:	-20.0v	T. Flow:	0.2ml/min
Sample	: CT8 LG-12	DL Temp:	250℃	B. conc:	50%H2O/50%ACN
Inj. Volume	: 1	Block Temp:	200℃		
MW	:1540.94				
Lot No.	:P200106-MJ772757				

REPORT -C8

Sample Description:

Structure : C8 LG-12

Number : 010250011

Lot No : P200106-MJ772757

Column : 4.6×250mm, Kromasil 100-5C18

Solvent A : 0.1% trifluoroacetic in 100% acetonitrile

Solvent B : 0.1% trifluoroacetic in 100% water

Gradient :

	A	B
0.01min	30%	70%
25min	55%	45%
25.1min	100%	0%
30min	STOP	

Flow rate : 1.0 mL/min

Wavelength : 220nm

Volume : 5ul

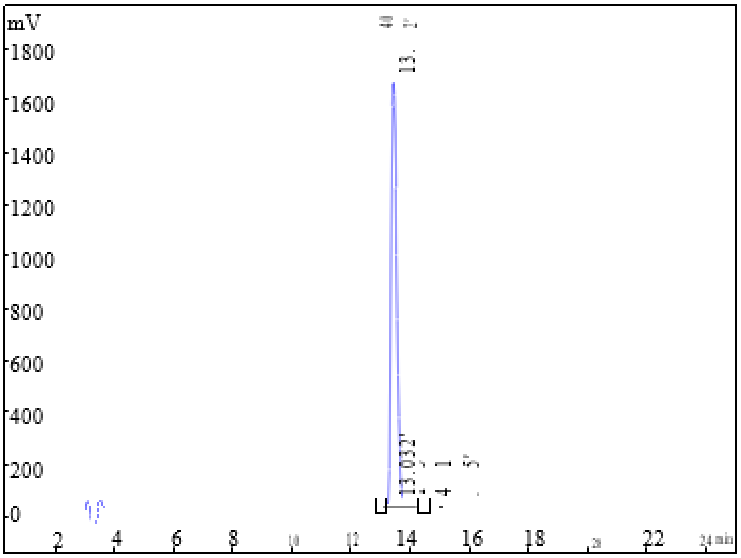

Rank	Time	Conc.	Area	Height
1	13.032	0.07746	15879	1457
2	13.402	99.74	20446309	1630245
3	14.515	0.1841	37739	2304
Total		100	20499927	1634006

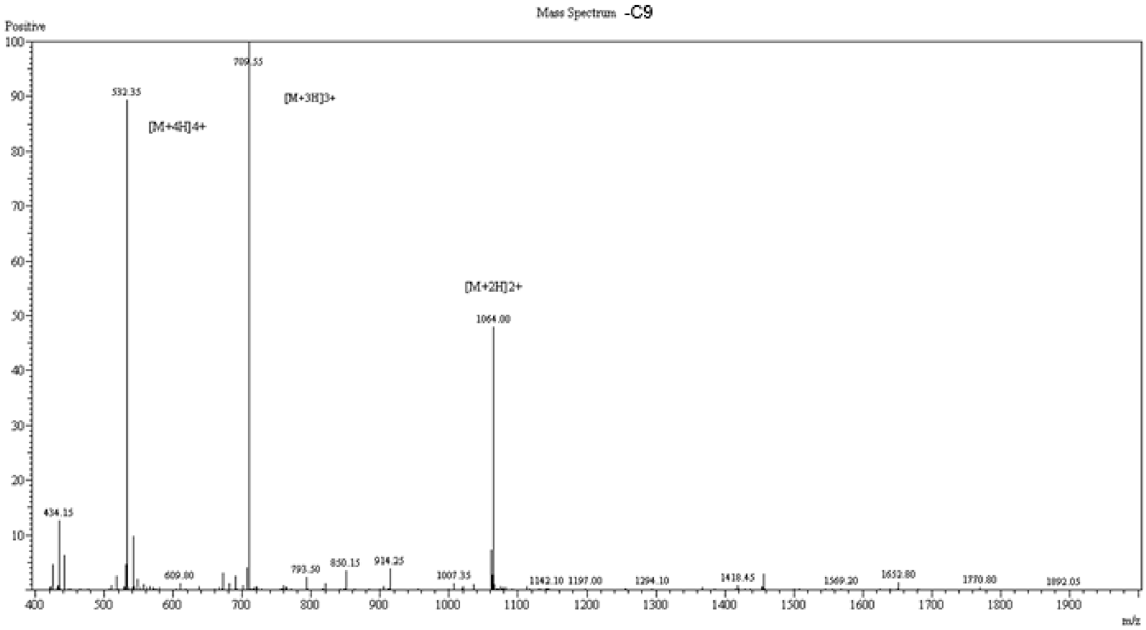

Sample Information

Date and Time	: 2020-1-13 12:23:10
User	: CHAO
Sample	: CT9 LR-16
Inj. Volume	: 1
MW	:2125.62
Lot No.	:P200106-MJ772758

Probe:	ESI	Probe bias:	-3.5kv
Nebulizer Gas Flow:	1.5L/min	Detector:	1.0kv
DL:	-20.0v	T. Flow:	0.2ml/min
DL Temp:	250℃	B. conc:	50%H2O/50%ACN
Block Temp:	200℃		

REPORT -C9

Sample Description:

Structure : C9 LR-16

Number : 010250011

Lot No : P200106-JQ772758

Column : 4.6×250mm, Kromasil 100-5C18

Solvent A : 0.1% trifluoroacetic in 100% acetonitrile

Solvent B : 0.1% trifluoroacetic in 100% water

Gradient :

	A	B
0.01min	20%	80%
25min	45%	55%
25.1min	100%	0%
30min	STOP	

Flow rate : 1.0 mL/min

Wavelength : 220nm

Volume : 5ul

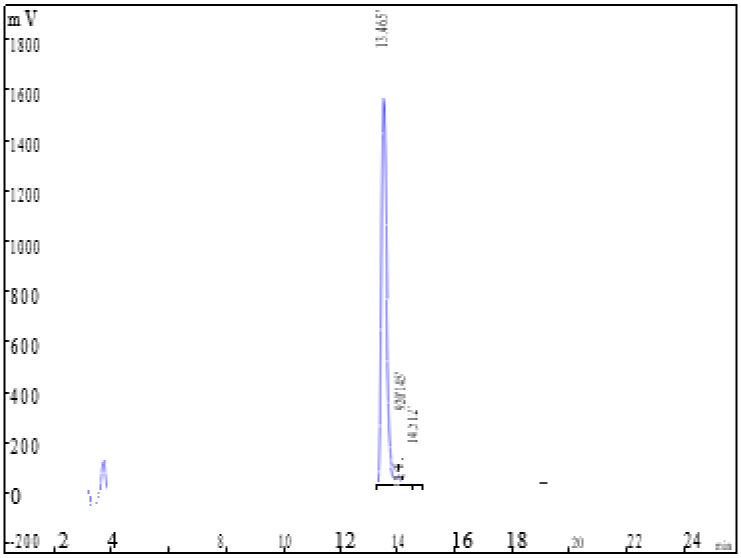

Rank	Time	Conc.	Area	Height
1	13.465	95.19	16909125	1530717
2	13.920	2.121	376811	37543
3	14.145	2.421	430065	39537
4	14.512	0.2707	48089	2330
Total		100	17764090	1610127

Mass Spectrum -C10

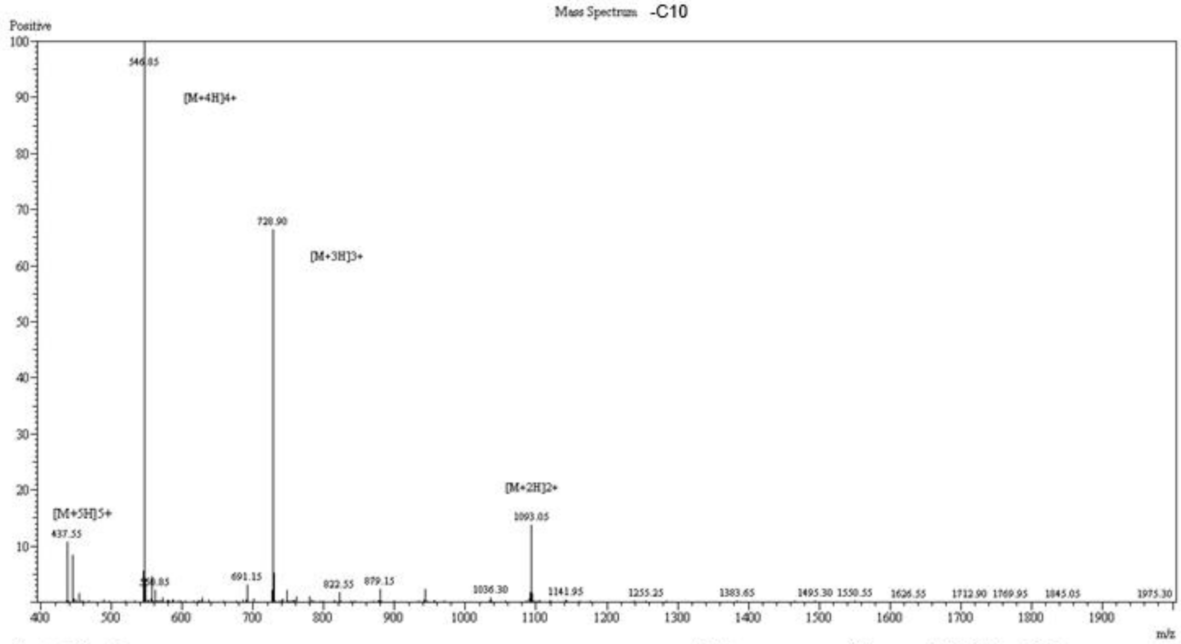

Sample Information		Probe:	ESI	Probe bias:	-3.5kv
Date and Time	: 2020-1-9 9:55:14	Nebulizer Gas Flow:	1.5L/min	Detector:	1.0kv
User	: CHAO	DL:	-20.0v	T. Flow:	0.2ml/min
Sample	: CT10 LR-16	DL Temp:	250℃	B. conc:	50%H2O/50%ACN
Inj. Volume	: 1	Block Temp:	200℃		
MW	:2183.66				
Lot No.	:P200106-MJ772759				

REPORT -C10

Sample Description:

Structure : C10 LR-16

Number : 010250011

Lot No : P200106-MJ772759

Column : 4.6×250mm, Kromasil 100-5C18

Solvent A : 0.1% trifluoroacetic in 100% acetonitrile

Solvent B : 0.1% trifluoroacetic in 100% water

Gradient :

	A	B
0.01min	23%	77%
25min	48%	52%
25.1min	100%	0%
30min	STOP	

Flow rate : 1.0 mL/min

Wavelength : 220nm

Volume : 5ul

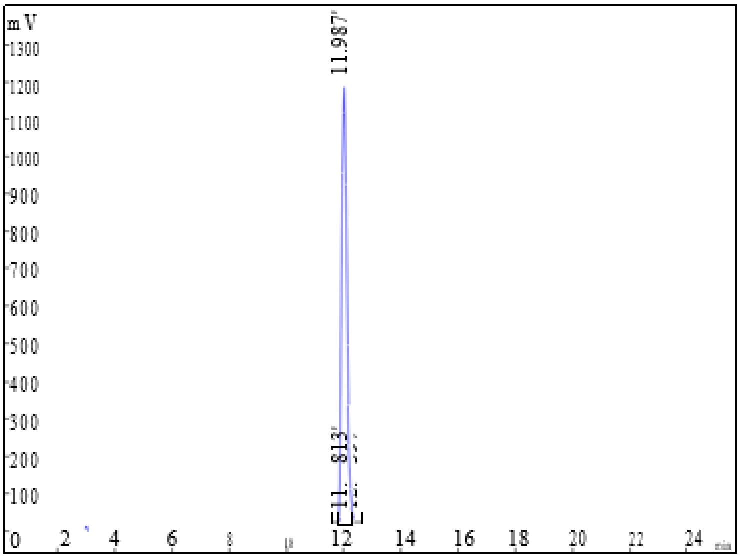

Rank	Time	Conc.	Area	Height
1	11.813	0.05078	6674	3423
2	11.987	99.88	13127402	1163632
3	12.397	0.06902	9071	2290
Total		100	13143147	1169345

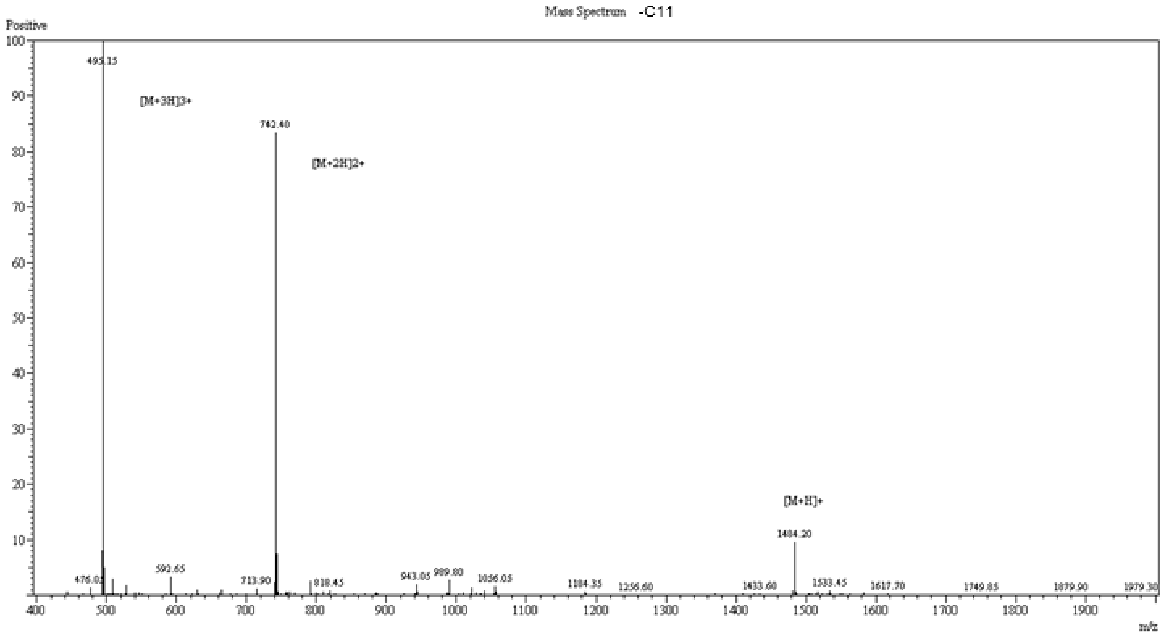

Sample Information	
Date and Time	: 2020-1-10 11:52:37
User	: CHAO
Sample	: CT11 LG-12
Inj. Volume	: 1
MW	:1482.90
Lot No.	:P200106-MJ772761

Probe:	ESI	Probe bias:	-3.5kv
Nebulizer Gas Flow:	1.5L/min	Detector:	1.0kv
DL:	-20.0v	T. Flow:	0.2ml/min
DL Temp:	250℃	B. conc:	50%H2O/50%ACN
Block Temp:	200℃		

REPORT -C11

Sample Description:

Structure : C11 LG-12

Number : 010250011

Lot No : P200106-MJ772761

Column : 4.6×250mm, Kromasil 100-5C18

Solvent A : 0.1% trifluoroacetic in 100% acetonitrile

Solvent B : 0.1% trifluoroacetic in 100% water

Gradient :

	A	B
0.01min	15%	85%
25min	40%	60%
25.1min	100%	0%
30min	STOP	

Flow rate : 1.0 mL/min

Wavelength : 220nm

Volume : 5ul

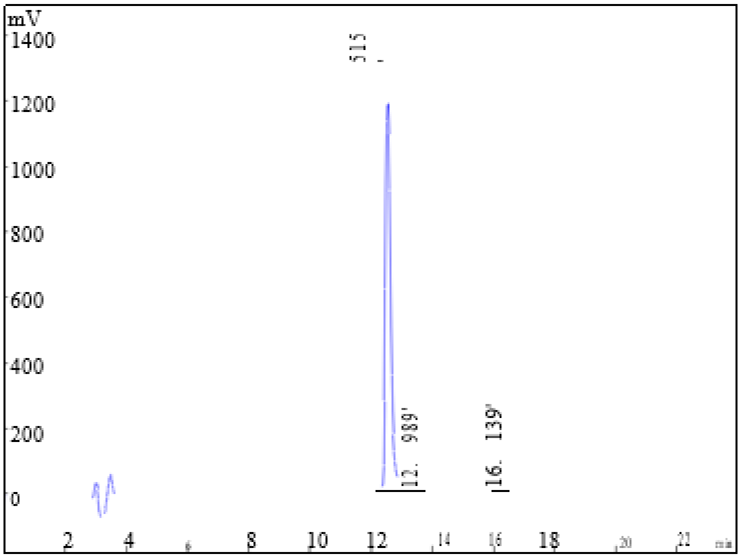

Rank	Time	Conc.	Area	Height
1	12.515	97.18	12279866	1171072
2	12.989	1.335	168682	16519
3	16.139	1.487	187850	16220
Total		100	12636398	1203811

Mass Spectrum -C12

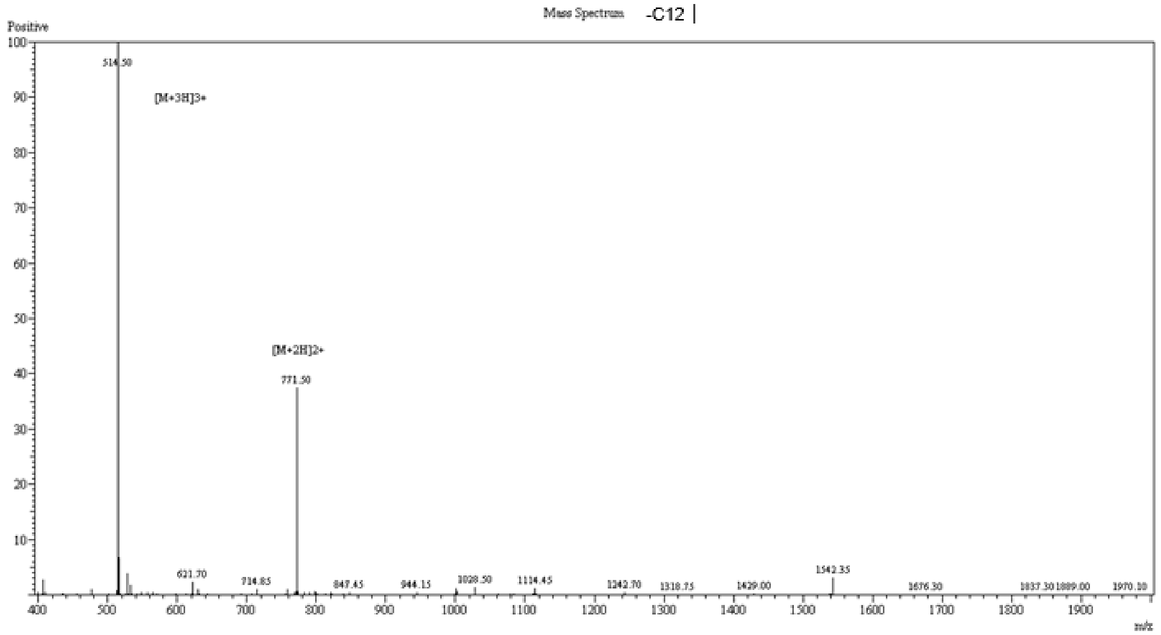

Sample Information		Probe:	ESI	Probe bias:	-3.5kv
Date and Time	: 2020-1-9 9:55:14	Nebulizer Gas Flow:	1.5L/min	Detector:	1.0kv
User	: CHAO	DL:	-20.0v	T. Flow:	0.2ml/min
Sample	: CT12 LG-12	DL Temp:	250℃	B. conc:	50%H2O/50%ACN
Inj. Volume	: 1	Block Temp:	200℃		
MW	:1540.94				
Lot No.	:P200106-MJ772762				

HPLC
REPORT -C12

Sample Description:

Structure : C12 LG-12
Number : 010250011
Lot No : P200106-MJ772762
Column : 4.6×250mm, Kromasil 100-5C18
Solvent A : 0.1% trifluoroacetic in 100% acetonitrile
Solvent B : 0.1% trifluoroacetic in 100% water

Gradient :	A	B
0.01min	23%	77%
25min	48%	52%
25.1min	100%	0%
30min	STOP	

Flow rate : 1.0 mL/min
Wavelength : 220nm
Volume : 5ul

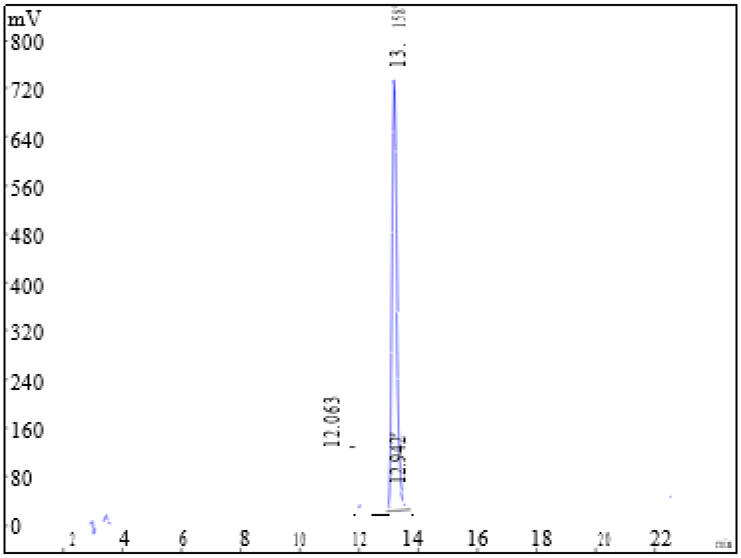

Rank	Time	Conc.	Area	Height
1	12.063	1.973	149559	15778
2	12.942	0.5075	38476	3629
3	13.158	97.52	7393997	711646
Total		100	7582032	731053

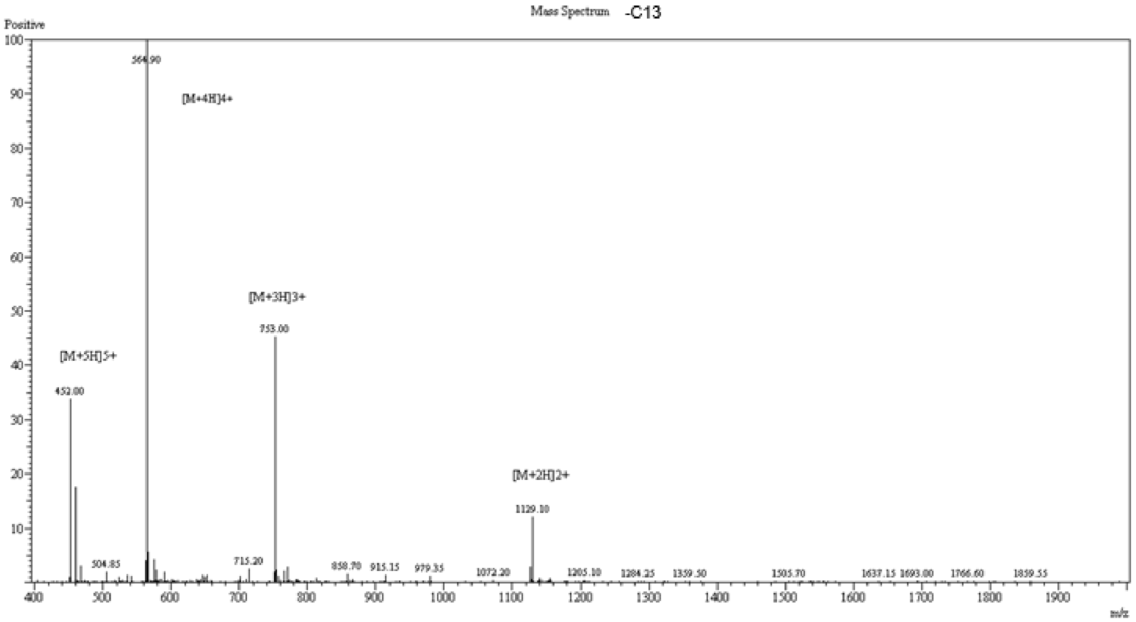

Sample Information		Probe:	ESI	Probe bias:	-3.5kv
Date and Time	: 2020-2-21 15:27:44	Nebulizer Gas Flow:	1.5L/min	Detector:	1.0kv
User	: CHAO	DL:	-20.0v	T. Flow:	0.2ml/min
Sample	: LR-16	DL Temp:	250℃	B. conc:	50%H2O/50%ACN
Inj. Volume	: 1	Block Temp:	200℃		
MW	:2255.9				
Lot No.	:P200211-MJ781299				

HPLC REPORT -C13

Structure : LR-16

Number : 010250011

Lot No : P200211-MJ781299

Column : 4.6×250mm, Kromasil 100-5C18

Solvent A : 0.1% trifluoroacetic in 100% acetonitrile

Solvent B : 0.1% trifluoroacetic in 100% water

Gradient :

	A	B
0.01min	25%	75%
25min	50%	50%
25.1min	100%	0%
30min	STOP	

Flow rate : 1.0 mL/min

Wavelength : 220nm

Volume : 5ul

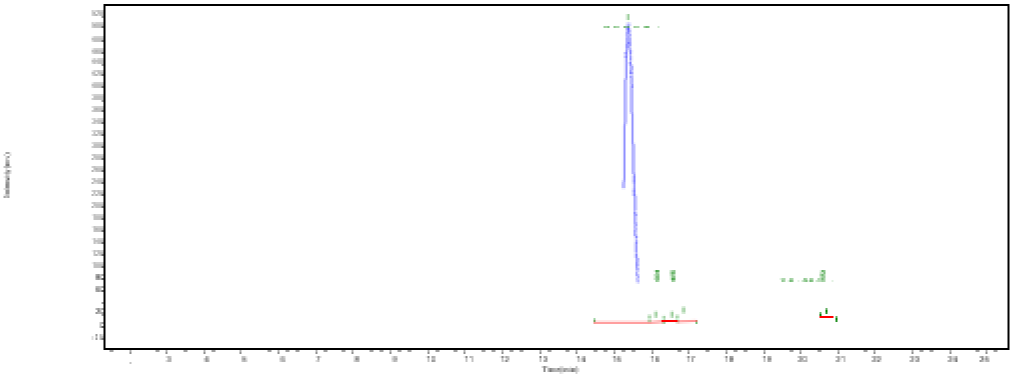

Peak No.	Ret Time	Height	Area	Conc.
1	15.372	505074.031	8256661.500	95.2012
2	16.133	6648.099	117775.125	1.3580
3	16.533	6634.114	104721.688	1.2075
4	16.863	13236.716	148107.047	1.7077
5	20.707	5123.034	45587.508	0.5256
Total				100.0000

Mass Spectrum -C14

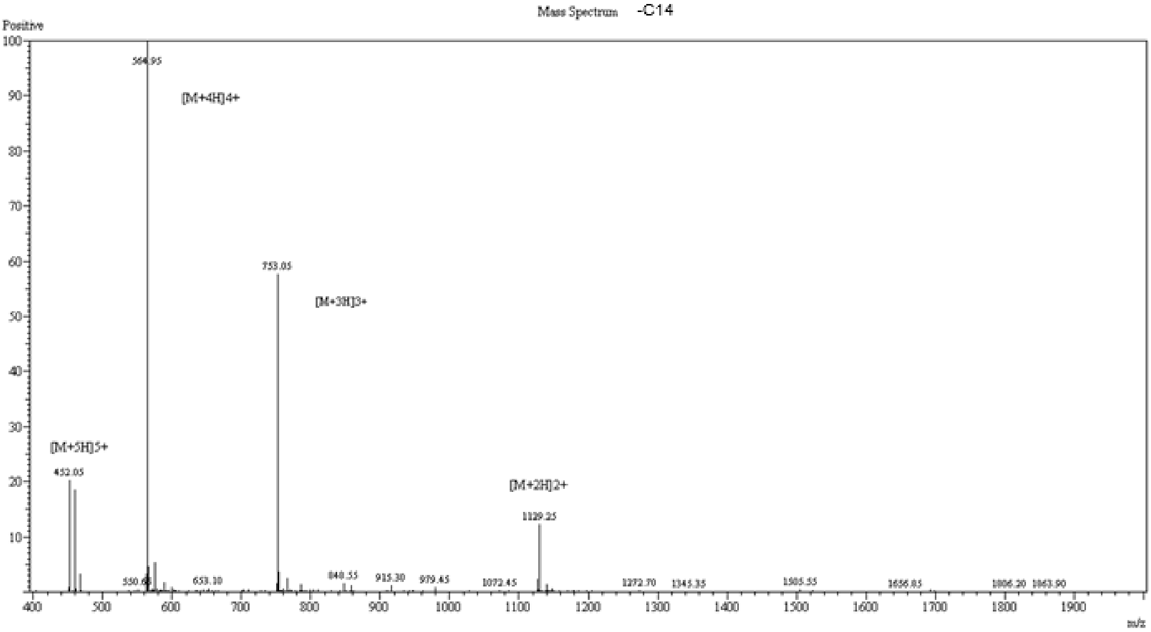

Sample Information	
Date and Time	: 2020-2-21 9:42:46
User	: CHAO
Sample	: LR-16
Inj. Volume	: 1
MW	:2255.90
Lot No.	:P200211-MJ781300

Probe:	ESI	Probe bias:	-3.5kv
Nebulizer Gas Flow:	1.5L/min	Detector:	1.0kv
DL:	-20.0v	T. Flow:	0.2ml/min
DL Temp:	250℃	B. conc:	50%H2O/50%ACN
Block Temp:	200℃		

HPLC -C14

Sample Description:
Analyst:YSC
Structure:LR-16
Lot NO.:P200211-MJ781300
Number:0200193
Column:250*4.6mm,Boston Green ODS-AQ
Solvent A:0.1%TFA in 100%water
Solvent B:0.1%TFA in 100%acetonitrile

Gradient :	A	B
0.1min	75	25
25min	50	50
25.01min	0	100
30min	0	100

Flow rate:1.0ml/min
Wavelength(nm):220
Volume:10ul
File opened: D:\2020 HPLC\LR-16-F 781300 200221.hw, where

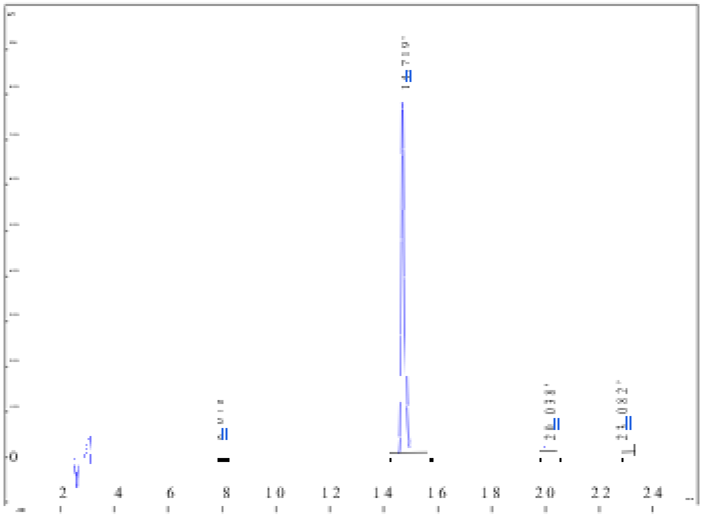

Rank	Time	Conc.	Area	Height
1	8.078	1.48	105399	11826
2	14.719	95.32	6787298	773221
3	20.038	2.09	148802	15692
4	23.082	1.109	78945	6547
Total		100	7120444	807286

Mass Spectrum -C15

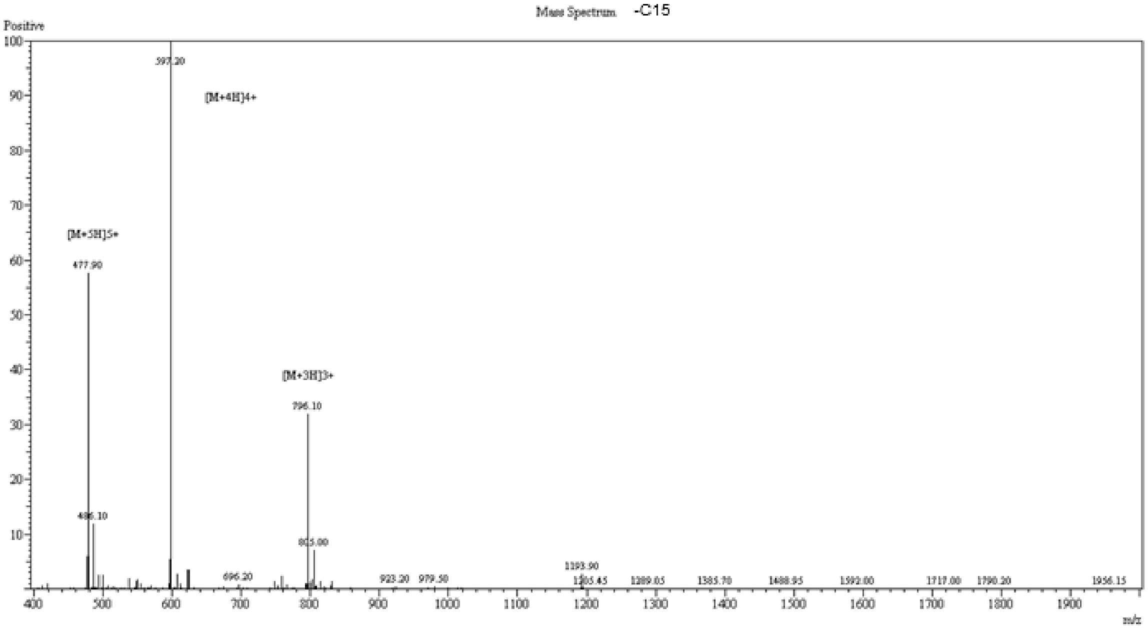

Sample Information	
Date and Time	: 2020-2-20 10:02:03
User	: CHAO
Sample	: LR-16
Inj. Volume	: 1
MW	:2385.05
Lot No.	:P200211-MJ781301

Probe:	ESI	Probe bias:	-3.5kv
Nebulizer Gas Flow:	1.5L/min	Detector:	1.0kv
DL:	-20.0v	T. Flow:	0.2ml/min
DL Temp:	250℃	B. conc:	50%H2O/50%ACN
Block Temp:	200℃		

HPLC
-C15

Sample Description:
Analyst:YSC
Structure:LR-16
Lot NO.:P200211-MJ781301
Number:0200193
Column:250*4.6mm,Boston Green ODS-AQ
Solvent A:0.1%TFA in 100%water
Solvent B:0.1%TFA in 100%acetonitrile

Gradient :	A	B
0.1min	72	28
25min	47	53
25.01min	0	100
30min	0	100

Flow rate:1.0ml/min
Wavelength(nm):220
Volume:10ul
File opened: D:\2020\LR-16-F 781301 200220.hw, where

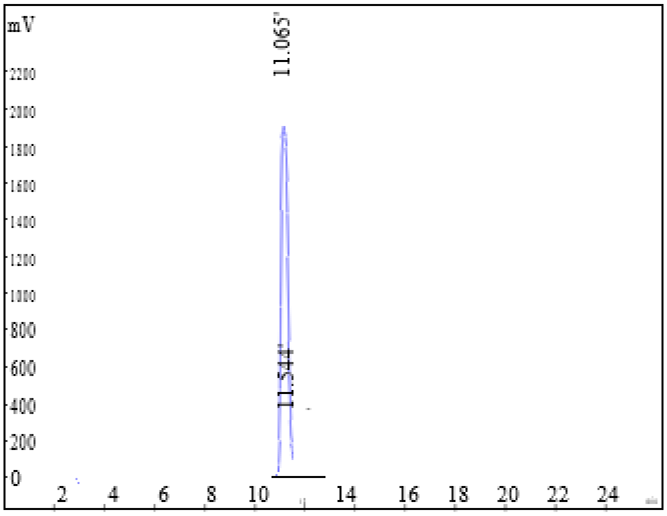

Rank	Time	Conc.	Area	Height
1	11.065	95.39	33221322	1891078
2	11.544	1.612	561229	42355
3	11.946	1.172	408038	24898
4	12.151	1.825	635470	22013
Total		100	34826059	1980344

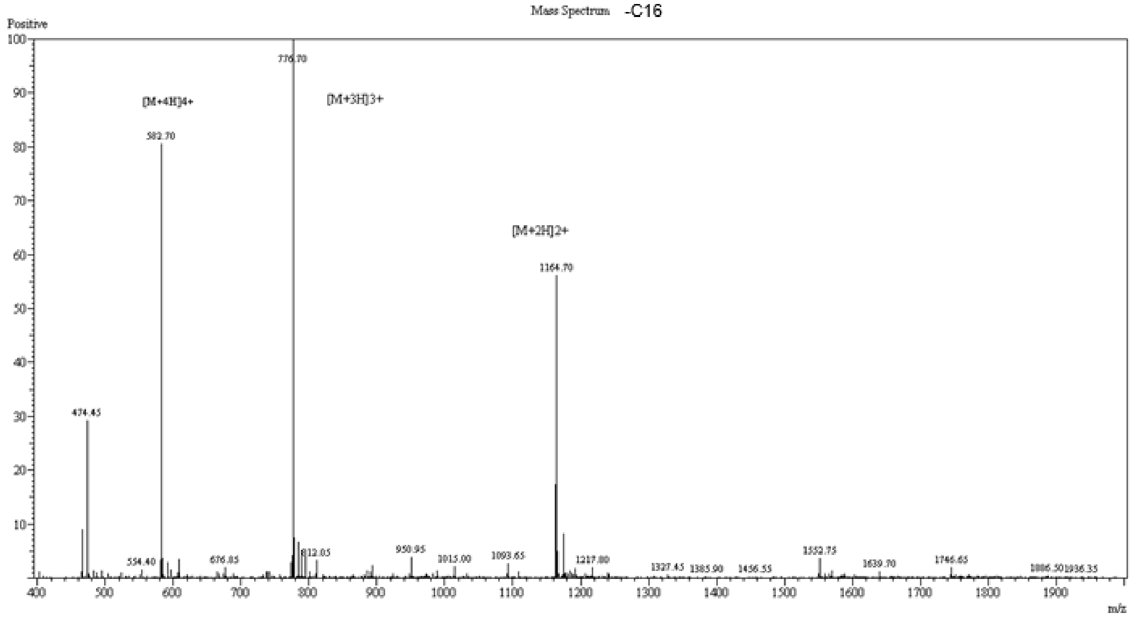

Sample Information	
Date and Time	: 2020-2-21 15:27:44
User	: CHAO
Sample	: LR-16
Inj. Volume	: 1
MW	:2327.02
Lot No.	:P200211-MJ781302

Probe:	ESI	Probe bias:	-3.5kv
Nebulizer Gas Flow:	1.5L/min	Detector:	1.0kv
DL:	-20.0v	T. Flow:	0.2ml/min
DL Temp:	250℃	B. conc:	50%H2O/50%ACN
Block Temp:	200℃		

HPLC REPORT -C16

Structure: LR-16

Number : 010250011

Lot No : P200211-MJ781302

Column : 4.6×250mm,Kromasil 100-5C18

Solvent A: 0.1% trifluoroacetic in 100% acetonitrile

Solvent B: 0.1% trifluoroacetic in 100% water

Gradient :	A	B
0.01min	20%	80%
25min	45%	55%
25.1min	100%	0%
30min	STOP	

Flow rate : 1.0 mL/min

Wavelength : 220nm

Volume : 5ul

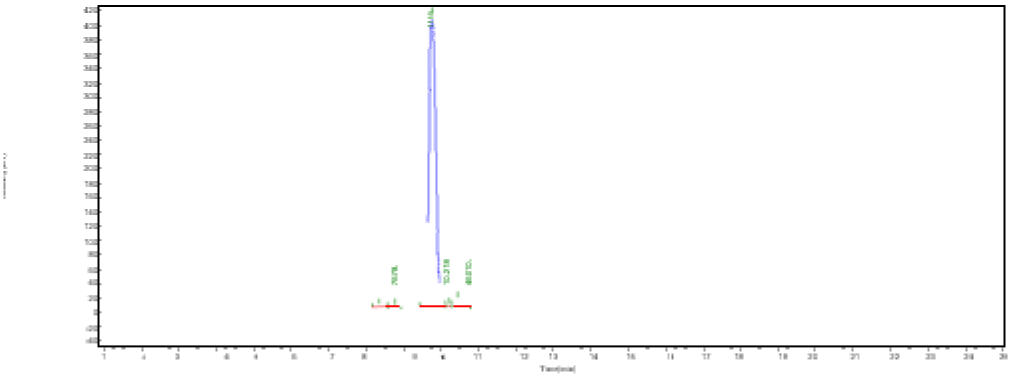

Peak No.	Ret Time	Height	Area	Conc.
1	8.347	3069.753	29357.186	0.5490
2	8.767	1570.017	15336.174	0.2868
3	9.775	409044.438	5160713.000	96.5070
4	10.218	3022.122	25842.918	0.4833
5	10.460	11414.246	116253.805	2.1740
Total				100.0000

Mass Spectrum -C17

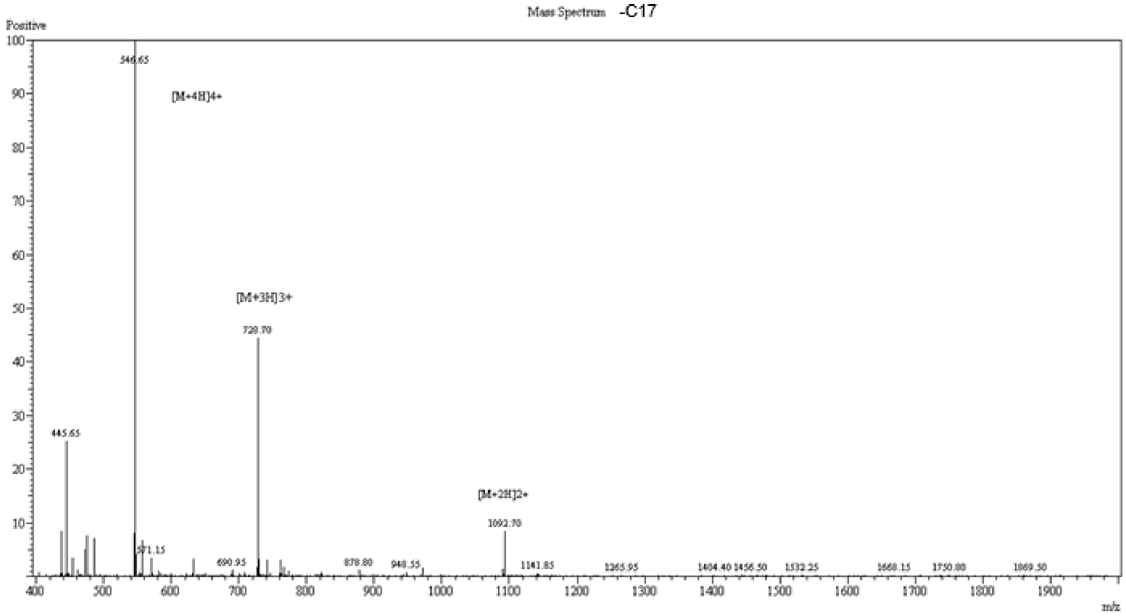

Sample Information						
Date and Time	: 2020-2-21 9:42:46		Probe:	ESI	Probe bias:	-3.5kv
User	: CHAO		Nebulizer Gas Flow:	1.5L/min	Detector:	1.0kv
Sample	: LR-16		DL:	-20.0v	T. Flow:	0.2ml/min
Inj. Volume	: 1		DL Temp:	250℃	B. conc:	50%H2O/50%ACN
MW	:2182.85		Block Temp:	200℃		
Lot No.	:P200211-MJ781303					

HPLC -C17

Sample Description:
Analyst:YSC
Structure:LR-16
Lot NO.:P200211-MJ781303
Number:0200193
Column:250*4.6mm,Boston Green ODS-AQ
Solvent A:0.1%TFA in 100%water
Solvent B:0.1%TFA in 100%acetonitrile

Gradient :	A	B
0.1min	80	20
25min	55	45
25.01min	0	100
30min	0	100

Flow rate:1.0ml/min
Wavelength(nm):220
Volume:10ul
File opened: D:\2020 HPLC\LR-16-F 781303 200221(20200221 20;51;59).hw, where

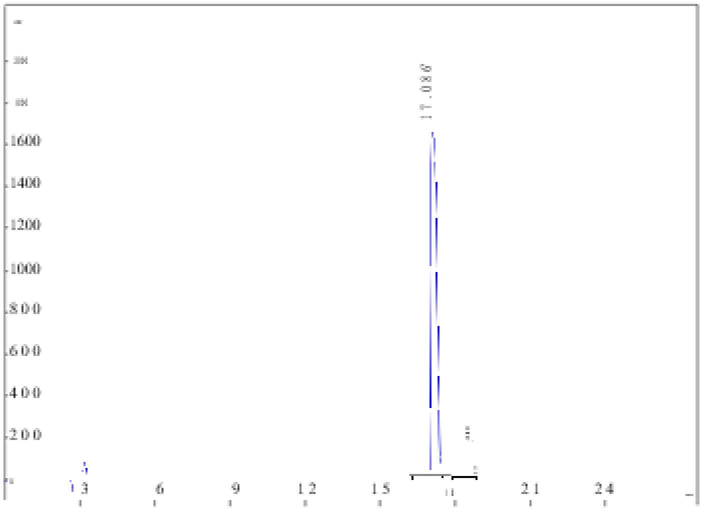

Rank	Time	Conc.	Area	Height
1	17.086	99.09	24899485	1636224
2	17.880	0.9064	227739	11594
Total		100	25127224	1647818

MASS SPECTROMETRY REPORT -C18

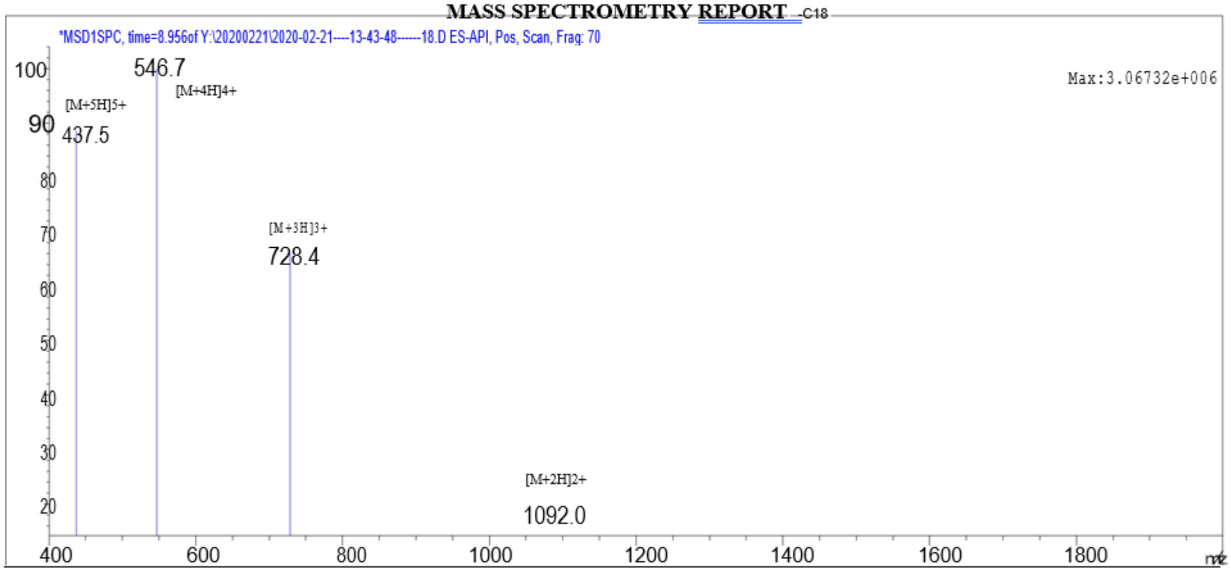

Sample Description		Instrument	Agilent-6125B		
Analyzed date:	2020-02-21	Probe:	ESI	Probe Bias:	+4.5kv
Analyst:	YU	Nebulizer Gas Flow:	1.5L/min	Detector:	1.5kv
Sample:	LR-16	CDL:	-20.0v	T. Flow:	0.2ml/min
M.W.:	2182.85	CDL Temp.:	250 ºC	B. Conc.:	50%H2O/50%ACN
Lot. No.:	P200211-MJ781304	Block Temp.:	200 ºC		

HPLC REPORT -C18

Structure : LR-16

Number : 010250011

Lot No : P200211-MJ781304

Column : 4.6×250mm,Sinochrom ODS-BP 5

Solvent A : 0.1% trifluoroacetic in 100% acetonitrile

Solvent B : 0.1% trifluoroacetic in 100% water

Gradient :	A	B
0.01min	28%	72%
25min	53%	47%
25.1min	100%	0%
30min	STOP	

Flow rate : 1.0 mL/min

Wavelength : 220nm

Volume : 5ul

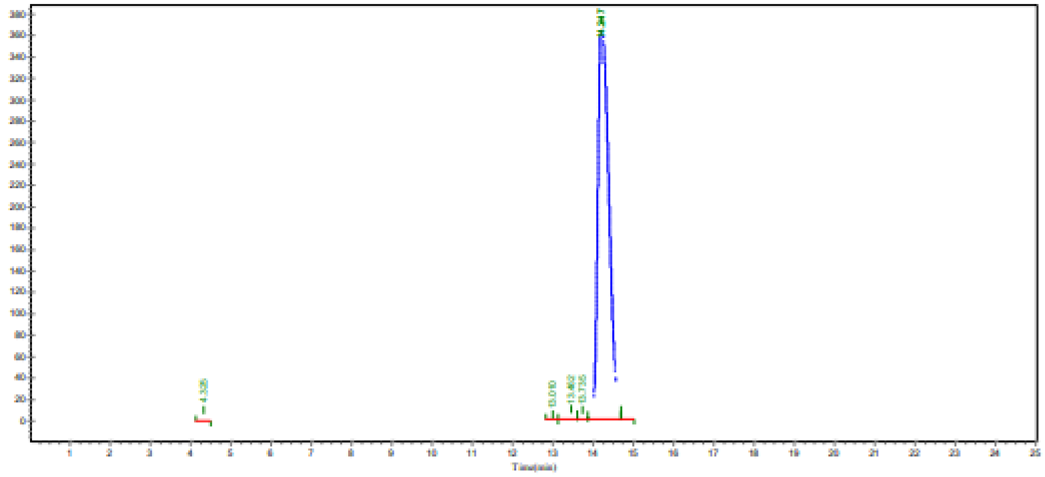

Peak No.	Ret Time	Height	Area	Conc..
1	4.325	6896.535	58696.102	0.8034
2	13.010	818.978	9135.670	0.1250
3	13.462	5990.662	89514.250	1.2253
4	13.735	5161.946	65366.742	0.8947
5	14.217	368961.938	7046807.500	96.4568
6	14.217	7317.516	36138.633	0.4947
Total				100.0000

Mass Spectrum -C19

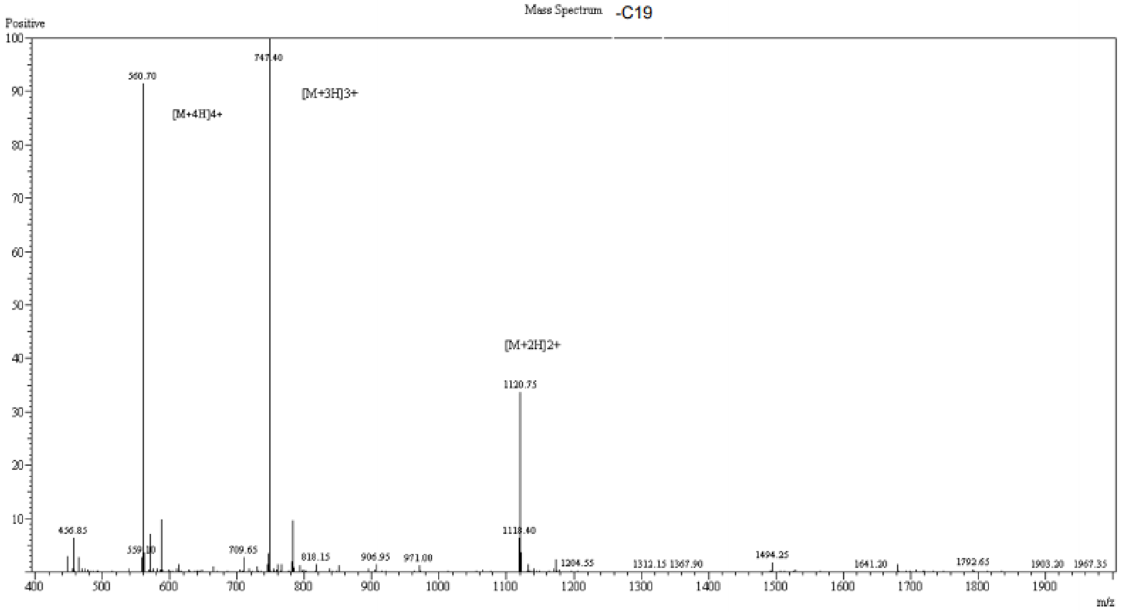

Sample Information		Probe:	ESI Probe bias: -3.5kv
Date and Time	: 2020-2-21 9:24:34	Nebulizer Gas Flow:	1.5L/min Detector: 1.0kv
User	: CHAO	DL:	-20.0v T. Flow: 0.2ml/min
Sample	: LR-16	DL Temp:	250℃ B. conc: 50%H2O/50%ACN
Inj. Volume	: 1	Block Temp:	200℃
MW	:2238.95		
Lot No.	:P200211-MJ781305		

HPLC REPORT -C19

Structure : LR-16
Number : 010250011
Lot No : P200211-MJ781305
Column : 4.6×250mm,Sinochrom ODS-BP 5
Solvent A : 0.1% trifluoroacetic in 100% acetonitrile
Solvent B : 0.1% trifluoroacetic in 100% water
Gradient :

	A	B
0.01min	37%	63%
25min	62%	38%
25.1min	100%	0%
30min	STOP	

Flow rate : 1.0 mL/min
Wavelength : 220nm
Volume : 5ul

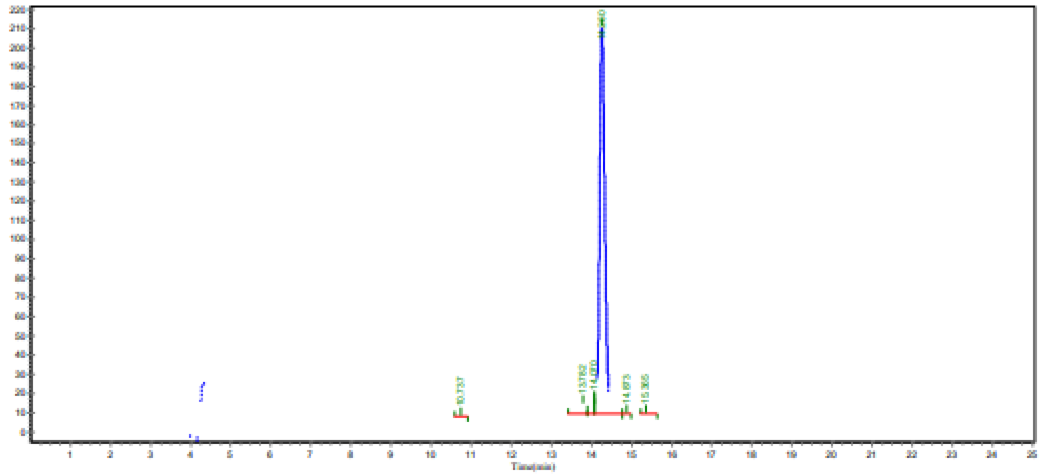

Peak No.	Ret Time	Height	Area	Conc..
1	10.737	379.749	2989.092	0.1562
2	13.782	5546.957	44716.340	2.3364
3	14.070	7557.202	35563.609	1.8582
4	14.260	201991.625	1824847.500	95.3487
5	14.873	438.334	2991.183	0.1563
6	15.365	382.416	2760.492	0.1442
Total				100.0000

Mass Spectrum -C20

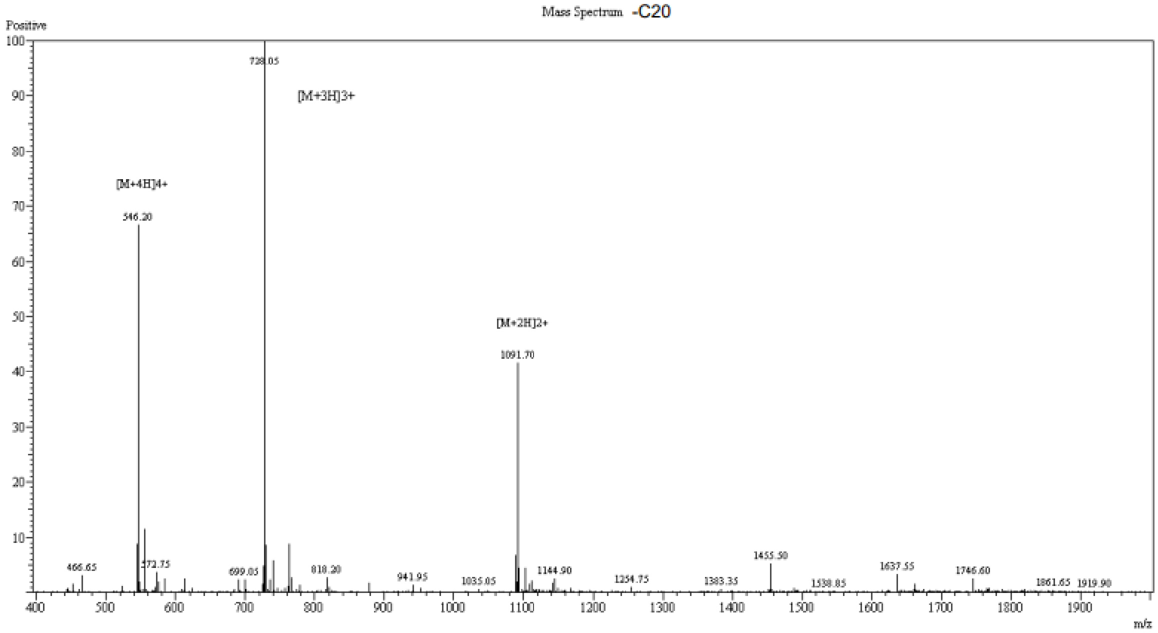

Sample Information	
Date and Time	: 2020-2-21 9:42:46
User	: CHAO
Sample	: LR-16
Inj. Volume	: 1
MW	:2180.92
Lot No.	:P200211-MJ781306

Probe:	ESI	Probe bias:	-3.5kv
Nebulizer Gas Flow:	1.5L/min	Detector:	1.0kv
DL:	-20.0v	T. Flow:	0.2ml/min
DL Temp:	250℃	B. conc:	50%H2O/50%ACN
Block Temp:	200℃		

HPLC -C20

Sample Description:
Analyst:YSC
Structure:LR-16
Lot NO.:P200211-MJ781306
Number:0200193
Column:250*4.6mm,Boston Green ODS-AQ
Solvent A:0.1%TFA in 100%water
Solvent B:0.1%TFA in 100%acetonitrile

Gradient :	A	B
0.1min	72	28
25min	47	53
25.01min	0	100
30min	0	100

Flow rate:1.0ml/min
Wavelength(nm):220
Volume:10ul
File opened: D:\2020\lr-16-f 781306 200221.hw, where

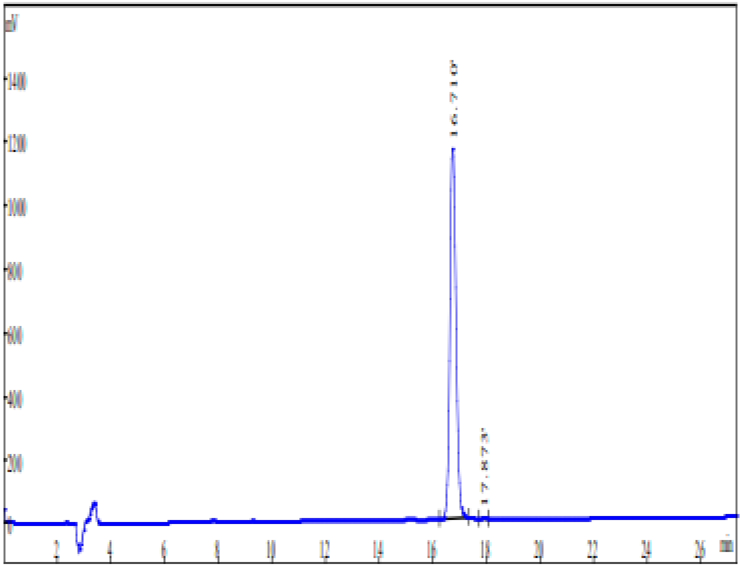

Rank	Time	Conc.	Area	Height
1	16.710	99.75	17259829	1164325
2	17.873	0.2534	43846	5362
Total		100	17303675	1169687

MASS SPECTROMETRY REPORT C21

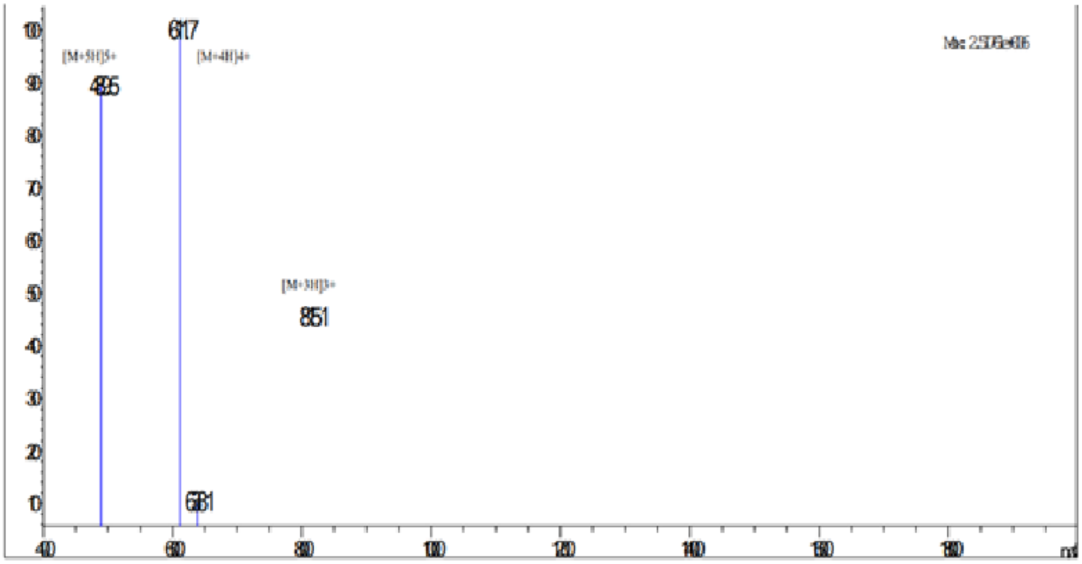

Sample Description		Instrument	Agilent-6125B		
Analyzed date:	2020-02-20	Probe:	ESI	Probe Bias:	+4.5kv
Analyst:	YU	Nebulizer Gas Flow:	1.5L/min	Detector:	1.5kv
Sample:	LR-16	CDL:	-20.0v	T. Flow:	0.2ml/min
M.W.:	2443.08	CDL Temp.:	250 °C	B. Conc.:	50%H2O/50%ACN
Lot. No.:	P200211-MJ781307	Block Temp.:	200 °C		

HPLC REPORT -C21

Structure : LR-16

Number : 010250011

Lot No : P200211-MJ781307

Column : 4.6×250mm,Sinochrom ODS-BP 5

Solvent A : 0.1% trifluoroacetic in 100% acetonitrile

Solvent B : 0.1% trifluoroacetic in 100% water

Gradient :	A	B
0.01min	32%	68%
25min	57%	43%
25.1min	100%	0%
30min	STOP	

Flow rate : 1.0 mL/min

Wavelength : 220nm

Volume : 5ul

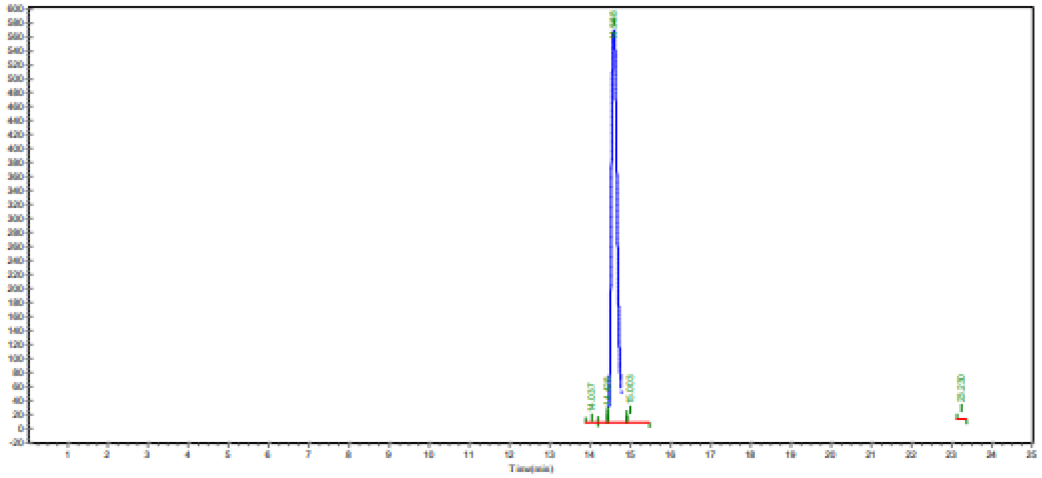

Peak No.	Ret Time	Height	Area	Conc..
1	14.037	1349.542	15164.871	0.2547
2	14.428	10651.179	57934.039	0.9730
3	14.588	566633.000	5682708.500	95.4367
4	15.003	11920.900	130646.016	2.1941
5	23.230	9561.931	67972.742	1.1415
Total				100.0000

MASS SPECTROMETRY REPORT -C22

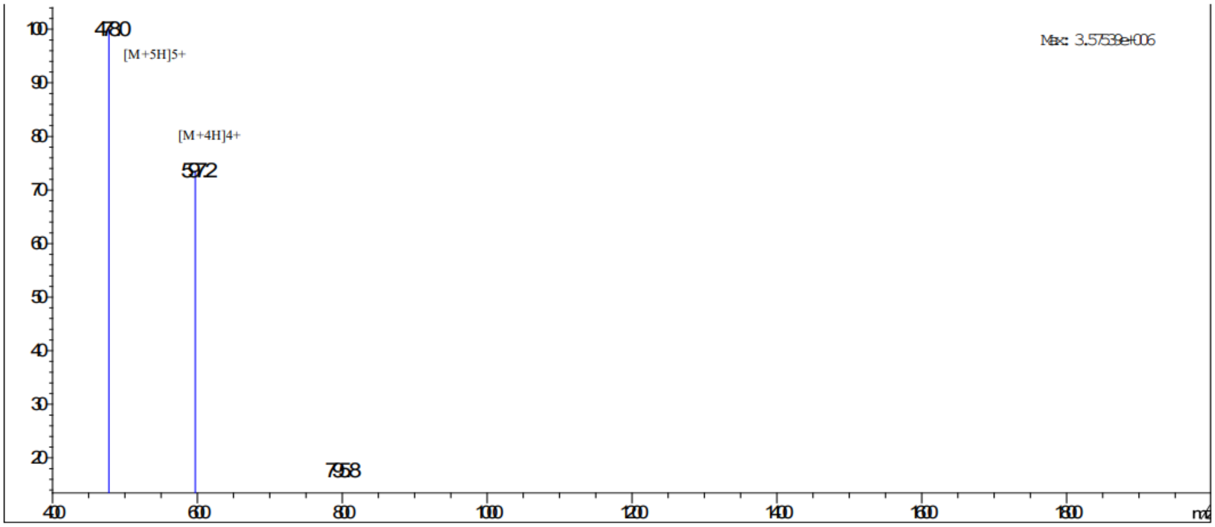

Sample Description		Instrument	Agilent-6125B		
Analyzed date:	2020-02-29	Probe:	ESI	Probe Bias:	+4.5kv
Analyst:	YU	Nebulizer Gas Flow:	1.5L/min	Detector:	1.5kv
Sample:	LR-16	CDL:	-20.0v	T. Flow:	0.2ml/min
M.W.:	2385.05	CDL Temp.:	250 °C	B. Conc.:	50%H2O/50%ACN
Lot. No.:	P200211-MJ781308	Block Temp.:	200 °C		

REPORT -C22

Sample Description:

Structure : LR-16
Number : 0200046
Analyst : HCM
Lot# : P200211-MJ781308
Column : 4.6mm*250mm, Venusil XBP-C18
Solvent A : 0.1% trifluoroacetic in 100% acetonitrile
Solvent B : 0.1% trifluoroacetic in 100% water

Gradient :

	A	B
0.01min	32%	68%
25min	57%	43%
25.1min	100%	0%
30.0min	STOP	

Flow rate : 1.0ml/min
Wavelength : 220nm
Volumn : 5ul

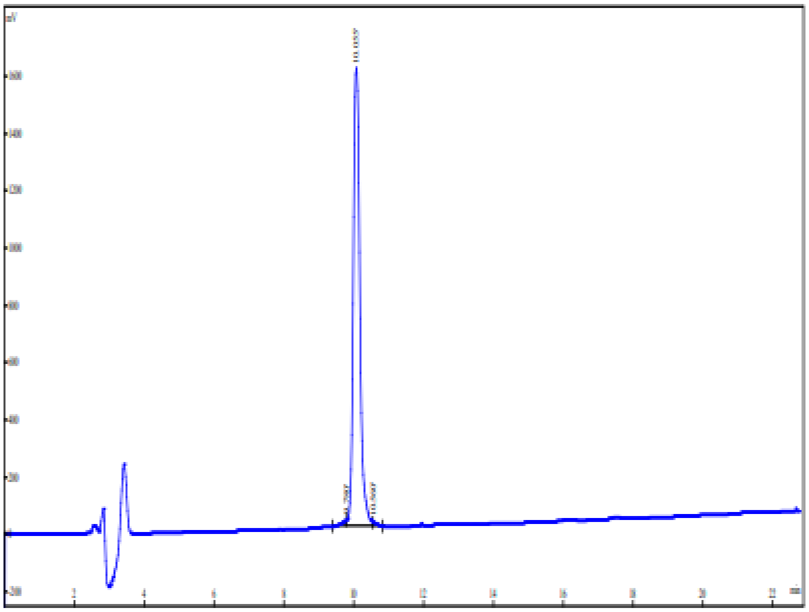

Rank	Time	Conc.	Area	Height
1	9.780	1.051	224028	23373
2	10.055	98.61	21029795	1603747
3	10.560	0.3382	72126	11790
Total		100	21325949	1638910

Mass Spectrum -C23

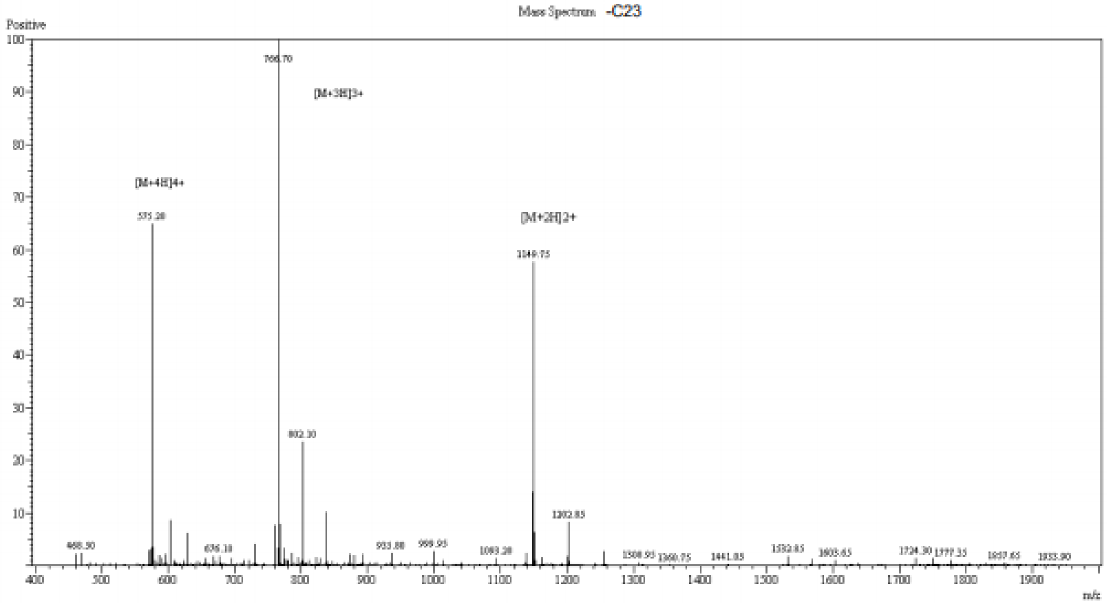

Sample Information		Probe:	ESI	Probe bias:	-3.5kv
Date and Time	: 2020-2-21 14:06:27	Nebulizer Gas Flow:	1.5L/min	Detector:	1.0kv
User	: CHAO	DL:	-20.0v	T. Flow:	0.2ml/min
Sample	: LR-16	DL Temp:	250℃	B. conc:	50%H2O/50%ACN
Inj. Volume	: 1	Block Temp:	200℃		
MW	:2296.98				
Lot No.	:P200211-MJ781309				

HPLC REPORT -C23

Structure : LR-16

Number : 010250011

Lot No : P200211-MJ781309

Column : 4.6×250mm,Kromasil 100-5C18

Solvent A : 0.1% trifluoroacetic in 100% acetonitrile

Solvent B : 0.1% trifluoroacetic in 100% water

Gradient :	A	B
0.01min	40%	60%
25min	65%	35%
25.1min	100%	0%
30min	STOP	

Flow rate : 1.0 mL/min

Wavelength : 220nm

Volume : 5ul

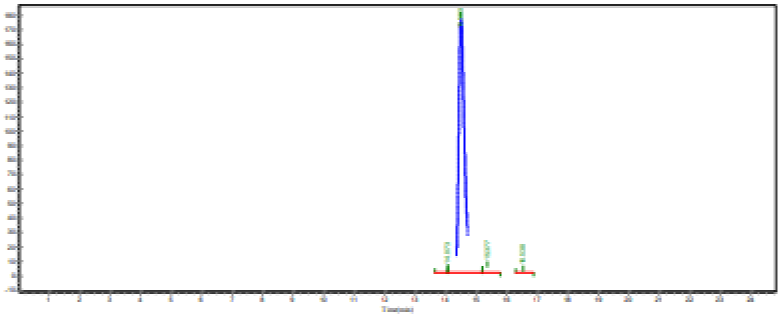

Peak No.	Ret Time	Height	Area	Conc.
1	14.073	1825.672	18094.828	0.7174
2	14.503	176059.781	2443543.500	96.8809
3	15.377	4032.513	50059.551	1.9847
4	16.538	723.222	10515.502	0.4169
Total				100.0000

Mass Spectrum -C24

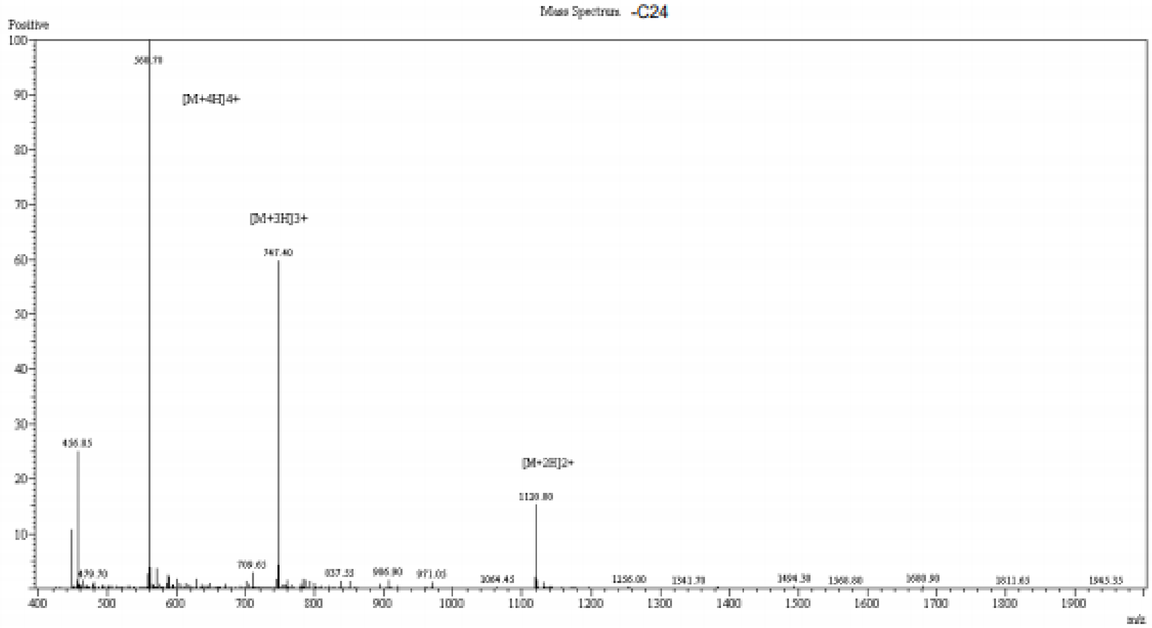

Sample Information		Probe:	ESI	Probe bias:	-3.5kv
Date and Time	: 2020-2-20 8:35:04	Nebulizer Gas Flow:	1.5L/min	Detector:	1.0kv
User	: CHAO	DL:	-20.0v	T. Flow:	0.2ml/min
Sample	: LR-16	DL Temp:	250℃	B. conc:	50%H2O/50%ACN
Inj. Volume	: 1	Block Temp:	200℃		
MW	:2238.95				
Lot No.	:P200211-MJ781310				

HPLC REPORT -C24

Structure : LR-16

Number : 010250011

Lot No : P200211-MJ781310

Column : 4.6×250mm,Kromasil 100-5C18

Solvent A : 0.1% trifluoroacetic in 100% acetonitrile

Solvent B : 0.1% trifluoroacetic in 100% water

Gradient :

	A	B
0.01min	33%	67%
25min	58%	42%
25.1min	100%	0%
30min	STOP	

Flow rate : 1.0 mL/min

Wavelength : 220nm

Volume : 5ul

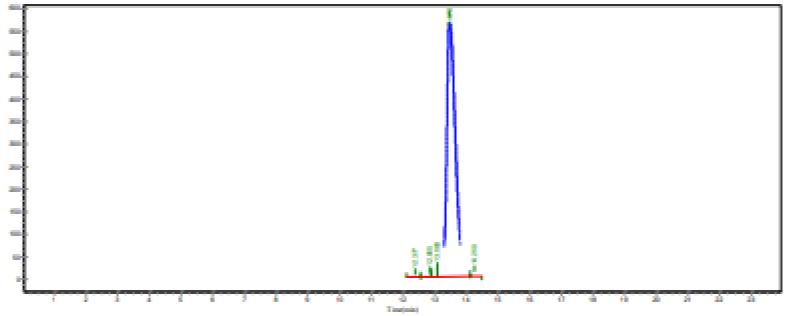

Peak No.	Ret Time	Height	Area	Conc.
1	12.377	5375.193	79238.063	0.7020
2	12.855	9943.389	137947.328	1.2222
3	13.083	19111.195	148997.516	1.3201
4	13.478	575532.500	10801205.000	95.6957
5	14.250	11149.404	119639.859	1.0600
Total				100.0000
